# Unmasking the Fungicidal Potency and Multifaceted Mechanisms of Nutmeg Essential Oil Against *Candida auris*

**DOI:** 10.3390/ph19020233

**Published:** 2026-01-29

**Authors:** Akriti Gaurav, Saif Hameed, Suhailah S. Aljameel, Suriya Rehman, Inès Hammami, Wissem Mnif, Zainab S. Alghamdi, Zeeshan Fatima

**Affiliations:** 1 Amity Institute of Biotechnology, Amity University Haryana, Manesar, Gurugram 122413, India; akritinarayan11@gmail.com (A.G.); saifhameed@yahoo.co.in (S.H.); 2 Department of Chemistry, College of Science, Imam Abdulrahman Bin Faisal University, Dammam 31441, Saudi Arabia; zsalghamdi@iau.edu.sa; 3 Department of Epidemic Diseases Research, Institute for Research and Medical Consultation (IRMC), Imam Abdulrahman Bin Faisal University, Dammam 31441, Saudi Arabia; surrehman@iau.edu.sa; 4 Department of Biology, College of Science, Imam Abdulrahman Bin Faisal University, Dammam 31441, Saudi Arabia; ihammami@iau.edu.sa; 5 Basic and Applied Scientific Research Center, Imam Abdulrahman Bin Faisal University, Dammam 31441, Saudi Arabia; 6 Department of Chemistry, College of Science, University of Bisha, Bisha 61922, Saudi Arabia; w_mnif@yahoo.fr; 7 Environmental and Sustainable Unit, Basic and Applied Scientific Research Center (BASRC), Imam Abdulrahman Bin Faisal University, Dammam 31441, Saudi Arabia

**Keywords:** *Candida auris*, essential oil, ergosterol, efflux pump, biofilm

## Abstract

**Background**: *Candida auris* has emerged as a multidrug-resistant fungal pathogen, presenting significant clinical challenges worldwide. Although considerable progress has been made in antifungal research, the specific mechanisms underlying drug resistance in *C. auris* remain incompletely understood. To overcome this problem, natural compounds can be used as valuable alternatives. The present study aimed to evaluate the antifungal activity of NEO against *C. auris* and to understand the functional mechanisms underlying its antifungal activity. **Methods**: The antifungal activity of NEO against *C. auris* strain CBS10913T was examined using broth microdilution and spot assays to determine the minimum inhibitory concentration (MIC) and minimum fungicidal concentration (MFC). Mechanistic investigations were performed using phenotypic-, biochemical-, and fluorescence-based assays to evaluate its effects on cell wall integrity, membrane permeability, efflux pump activity, oxidative stress, lipid peroxidation, biofilm formation, and host cell adherence. Hemolytic assays were performed to evaluate preliminary biocompatibility. **Results**: During our study, we found that NEO showed strong fungicidal activity against *C. auris,* with an MIC of 500 µg/mL and an MFC of 650 µg/mL, and disrupted fungal cell wall integrity, significantly reduced ergosterol content, and inhibited efflux pump activity, leading to increased accumulation of fluorescent substrates. NEO induced increased intracellular reactive oxygen species, leading to oxidative-mediated lipid peroxidation and DNA damage. Moreover, NEO also suppressed stress biofilm formation, reduced metabolic activity, and decreased adherence to buccal epithelial cells, and it showed negligible hemolytic activity up to 2× MIC, indicating preliminary biocompatibility. **Conclusions**: This study demonstrates that NEO utilizes broad antifungal activity through multiple functional and phenotypic mechanisms, including disruption of membrane integrity, inhibition of efflux pump, induction of oxidative stress, and suppression of biofilm formation. Although the direct effects on pathogenicity-related genes or proteins were not studied, the findings still show NEO as a promising natural antifungal agent.

## 1. Introduction

The emergence of *Candida. auris* as a multidrug-resistant fungal pathogen presents a major challenge to global healthcare systems due to its high mortality rates and resistance to conventional antifungals [1,2,3]. *C. auris*, first reported in 2009, has rapidly become a serious concern to global health due to its multidrug resistance and high mortality in susceptible populations [4,5]. It has been associated with severe bloodstream infections, as well as a range of other hospital-acquired infections, like meningitis, bone infections, surgical site infections, and urinary tract infections [6]. It can survive on medical equipment and environmental surfaces for months and tends to colonize patients’ skin more readily than other mucosal areas, which greatly increases the risk of direct human-to-human transmission [7]. *C. auris* is notable for its ability to acquire resistance to all three main classes of antifungal agents. Its biofilm-forming ability further increases resistance to antifungal treatments, a trait shared across all *C. auris* strains and clades [8,9]. Resistance to azoles, echinocandins, and polyenes is frequently mediated by mechanisms such as increased efflux pump activity, alterations in ergosterol biosynthesis, and biofilm-associated protection, thereby creating a need to develop and explore new novel antifungal agents with multi-targeted modes of action [10,11]. Generally, the clinical management of *Candida* infections involves checking basic systemic conditions, maintaining oral hygiene, and applying topical antifungal agents [12,13,14]. Systemic antifungal treatment is typically reserved for cases that fail to respond to initial treatment or for recurrent infections [15,16]. However, current therapies are limited by toxicity, resistance, and poor efficacy against biofilms, highlighting the need for alternative strategies to treat *C. auris* infections [12]. Natural products, including a wide variety of plant-derived essential oils, have attracted considerable attention as potential antifungal agents due to their multiple bioactive constituents and safety [17]. Essential oils are composed of volatile compounds obtained from different plant materials, such as leaves, flowers, and bark primarily through steam distillation. Owing to their specific chemical compositions, they have also been widely used in aromatherapy. In addition, their volatile constituents have been tested for antimicrobial activity against different bacteria and fungi, including *C. auris* [7].

NEO, extracted from *Myristica fragrans*, mainly consists of monoterpene hydrocarbons such as sabinene, α-pinene, β-pinene, and D-limonene, along with the oxygenated monoterpene terpinen-4-ol. This composition was confirmed by GC-MS analysis in this study and in earlier research [18,19,20]. Recent studies have shown the efficacy of essential oil components against *Candida* species by targeting cell membrane integrity, efflux pumps, ergosterol biosynthesis, and biofilm formation [21,22]. For example, cinnamaldehyde components have been reported to inhibit *C. auris* growth by reducing ergosterol content and efflux pump activity, leading to membrane damage and ROS accumulation [22]. Similarly, antimicrobial peptides like dermaseptin trigger oxidative stress and lipid peroxidation in *C. auris* by disrupting membrane integrity without mitochondrial system failure [23].

Despite these advancements, the antifungal potential of NEO against *C. auris* remains poorly explored. Previous studies have reported that *Myristica fragrans* can inhibit the growth and morphogenesis of *Candida albicans* and can also act synergistically with conventional antifungal drugs [24]. In the present study, we investigated the antifungal potential and mechanisms of NEO against *C. auris*. By understanding these mechanisms, we aim to establish NEO as an alternative treatment option for global challenge posed by *C. auris* infections.

## 2. Results

### 2.1. Fungicidal Activity of NEO Against C. auris

**Figure 1:**
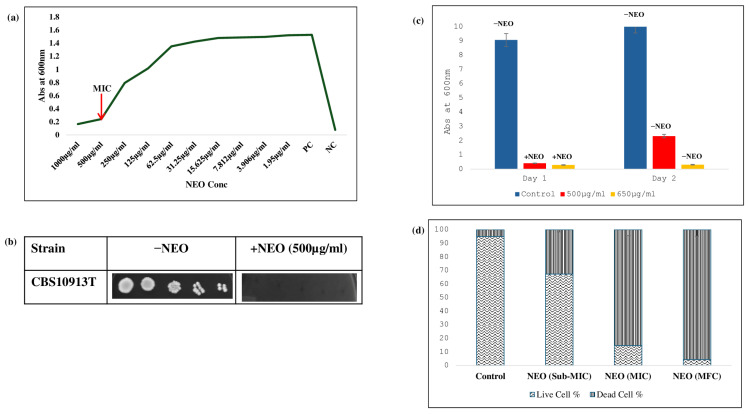
Antifungal activity of NEO: (**a**) broth microdilution assay to determine the MIC_90_ of *C. auris* in the presence of NEO. (**b**) Bar graph depicting the revival of untreated *C. auris* cells (−NEO) and non-revival of NEO-treated cells in YEPD media. (**c**) Spot assay of *C. auris* in the absence (−NEO) and presence of NEO (+NEO). (**d**) Cell viability assay of NEO. Bar graph depicting the cell viability at Sub-MIC, MIC, and MFC of NEO.

To determine the antifungal activity of NEO against *C. auris*, a broth microdilution method was used. It inhibited the growth of *C. auris* at a concentration of 500 μg/mL (Figure 1a). This result is supported by a spot assay, which showed the consistent results with the microdilution method (Figure 1b). To determine whether NEO is fungistatic or fungicidal in nature, a spectrophotometric growth assay was conducted where cultures were treated with NEO at different concentrations, and it showed complete growth inhibition on day one at 650 μg/mL, and no regrowth occurred when transferred to fresh medium without NEO on day two, which confirms the fungicidal activity of NEO at 650 μg/mL (Figure 1c). For biochemical and phenotypic analysis, a subinhibitory dose of 200 μg/mL was decided on, which inhibits growth and enables us to evaluate the effect of NEO on *C. auris*. Cell viability was further performed using grid analysis to quantify live and dead cells under different treatment conditions. Cells of the control cultures were almost viable, whereas at sub-MIC concentrations, we found that the viability of the cells reduced. In contrast, treatment at MIC and MFC concentrations caused cell death, showing the fungicidal nature of NEO (Figure 1d).

### 2.2. Impact of NEO on C. auris Cell Wall Composition

**Figure 2:**
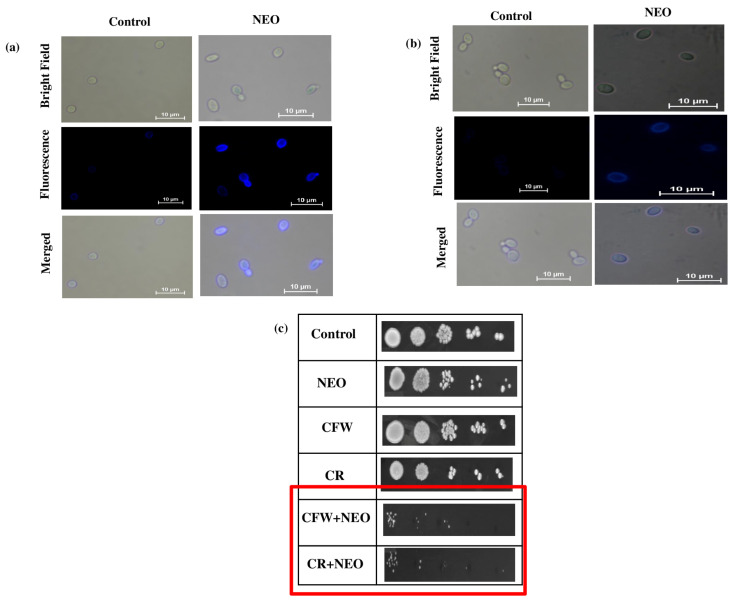
Effect of NEO on cell wall: (**a**) chitin (CFW staining) scale bar = 10 µm, (**b**) β1,3-glucan (AB staining) scale bar = 10 µm. (**c**) NEO-induced sensitivity in the fungal cell wall. Spot assay of control and NEO in presence of CR (10 μ mL) and CFW (10 μg/g/mL).

To study the effect of NEO on the cell wall of *C. auris*, fluorescent staining was used to visualize the polysaccharide components. Cells treated with NEO showed increased fluorescence intensity of CFW, a chitin-specific probe, compared with the untreated control (Figure 2a). Similarly, aniline blue staining for β-1,3-glucan showed high fluorescence in cells treated with NEO (Figure 2b). Further, to check the cell wall integrity, spot assays were performed on media containing cell wall stressors CFW and Congo Red. *C. auris* cells treated with NEO showed sensitivity against both the dyes, with no colony formation compared to untreated controls (Figure 2c).

### 2.3. Disruption of Ergosterol Biosynthesis in C. auris by NEO

**Figure 3:**
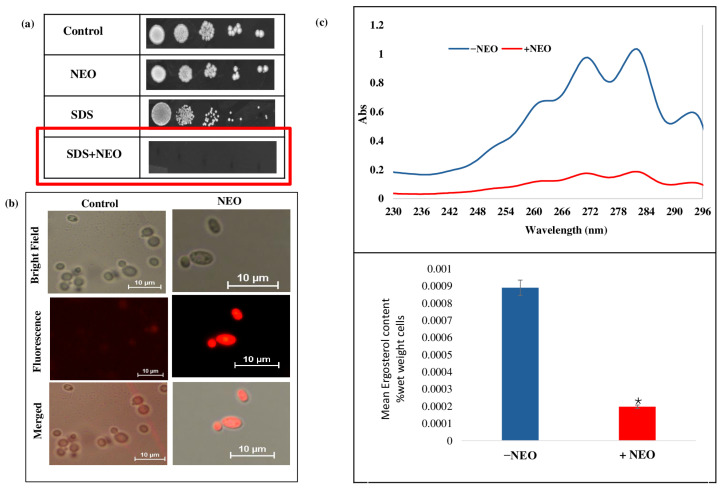
Effect of NEO on the membrane integrity. (**a**) Spot assay in the absence and presence of NEO with membrane damaging agent SDS (0.02%). (**b**) Propidium iodide (PI) staining of *C. auris* cells in the presence and absence of NEO. (**c**) Upper panel showing UV spectrophotometric ergosterol profiles of *C. auris* scanned between 220 to 300 nm in the absence and presence of NEO. Lower panel showing relative percentages of ergosterol content under the same conditions. Values are normalized to the untreated control, set at 100%, and expressed as mean ± standard deviation from three independent experiments (* depicts *p* < 0.05).

To ascertain the impact of NEO on *C. auris* cell membrane integrity and composition, a second phenotypic assay and biochemical assays for membrane stability and sterol levels were performed. Spot dilution assays on media containing (0.02% *w*/*v*) SDS revealed a strong hypersensitivity of NEO-treated cells when compared with the untreated control (Figure 3a), indicating a defective membrane barrier function. Visualized by fluorescence assay, increased fluorescence intensity was observed in the cells treated with NEO at MIC concentration compared to untreated control cells (Figure 3b), demonstrating enhanced membrane permeability. Ergosterol, as a major component of the fungal cytoplasmic membrane, was quantified with UV spectrophotometry at 230–300 nm. The content of ergosterol in the *C. auris* cells was approximately 78% lower among those treated with NEO than in the untreated control (Figure 3c).

### 2.4. Suppression of Efflux Pump Activity in C. auris by NEO

**Figure 4:**
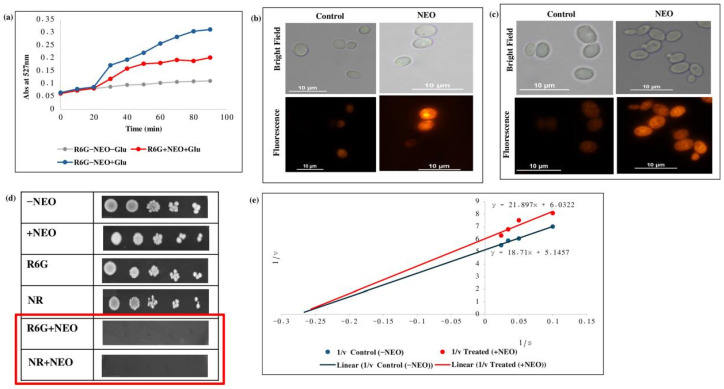
Effect of NEO on efflux pump activity. (**a**) Extracellular R6G concentrations in control and NEO-treated *C. auris* cells. Energy-dependent efflux of R6G was triggered by the addition of 2% glucose and quantified by measuring the absorbance of the supernatant at 527 nm. Data represent the mean ± standard deviation from three independent experiments, with error bars indicating variability. (**b**) R6G accumulation depicted by fluorescence images of R6G staining in the presence of NEO. Scale bar = 10 µm. (**c**) Nile red accumulation depicted by fluorescent images of Nile red staining in the presence of NEO. Scale bar = 10 µm. (**d**) Phenotypic susceptibility assay of R6G and Nile red in the presence and absence of NEO. (**e**) Lineweaver–Burk plot in the presence of NEO. The x-axis (1/S) represents the various concentrations (μM) of R6G used, and the y-axis (1/V) shows the rate of release of R6G in the presence of NEO.

Some additional experiments were also performed to investigate the effect of NEO on membrane-mediated efflux function in *C. auris* using a fluorescence-based efflux assay. The effect of R6G and Nile red as pump substrates was tested using the fluorescence-based and biochemical assays. Efflux assays revealed a strong loss of R6G efflux in NEO-treated cells versus untreated control, reflecting the level of transporter activity (Figure 4a). Fluorescence-based accumulation assays showed significantly higher intracellular fluorescence of R6G and Nile red in cells treated with NEO compared to the control, demonstrating the inhibition of efflux-pump activity (Figure 4b,c). Spot dilution assays showed that the addition of NEO to R6G and Nile red caused a greater degree of growth inhibition, as reflected by no colony formation versus the control (Figure 4d). Kinetic analysis of substrate transport plotted using Lineweaver–Burk representations showed a decrease in the apparent maximal transport velocity (Vmax) but no significant change in the apparent affinity (Km) after NEO challenge (Figure 4e). These changes are phenomenological and could result from transporter dysfunction due to modulations of membrane properties or lipid dynamics, rather than direct inhibition of the efflux transporter.

### 2.5. NEO Induces Oxidative Stress-Mediated Lipid Peroxidation and DNA Damage in C. auris

**Figure 5:**
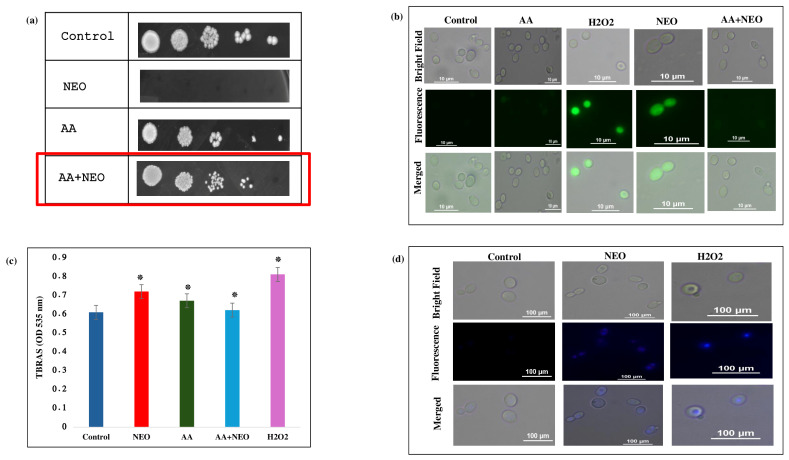
Effect of NEO on reactive oxygen species. (**a**) Spot assays showing hypersensitivity of *C. auris* cells in the presence of NEO at MIC alleviated by AA (20 mM), which is an antioxidant. (**b**) DCFDA staining of *C. auris* cells in the presence of NEO at MIC alleviated by AA (20 mM). (**c**) TBARS/MDA level as a measure of lipid peroxidation depicted as bar graph in the presence of NEO, AA (antioxidant) and H_2_O_2_ (oxidant). Mean of OD_570_ nm ± SD of three dependent sets of experiments are depicted on Y-axis and * depicts *p* < 0.05. (**d**) DAPI staining of *C. auris* cells in the presence of NEO.

To verify the role of NEO in causing oxidative stress and its related consequences for *C. auris*, a series of phenotypic and biochemical experiments were performed using *C. auris* with and without antioxidant supplementation. Spot dilution assays (Figure 5a) showed a significant growth inhibition of *C. auris* at the MIC value of NEO, while co-treatment with AA (10 mM) restored cell viability. In contrast, intracellular ROS levels, which were quantified by the DCFDA fluorescence intensity in NEO-treated cells, were significantly higher (Figure 5b) than those in untreated controls and less pronounced when compared to H_2_O_2_ (5 mM). When AA was with NEO, DCFDA fluorescence significantly decreased. Furthermore, the TBARS assay was used to evaluate downstream consequences of oxidative stress by lipid peroxidation. NEO treatment resulted in significantly higher levels of TBARS (Figure 5c), indicating increased membrane lipid damage, and H_2_O_2_-treated cells had the highest peroxidation levels. Co-treatment of AA reduced TBARS accumulation caused by NEO in cells, thus demonstrating the role of ROS in membrane damage. Further, from the DAPI staining, a bright fluorescence of the nuclei was observed in NEO/H_2_O_2_-treated cells compared to the control, indicating DNA damage due to oxidative stress (Figure 5d).

### 2.6. NEO Inhibits C. auris Biofilm Formation and Cell Adherence

**Figure 6:**
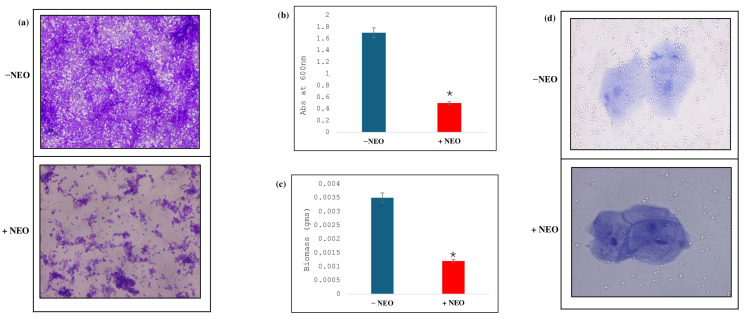
Effect of NEO on biofilm formation. (**a**) CV staining, showing the biofilm formation in absence (−NEO) and presence of NEO (+NEO). (**b**) MTT assay depicting the disrupted biofilm formation in NEO-treated cells. Mean of O.D_600_ nm ± SD of three independent sets of experiments is depicted on the y-axis and * depicts *p* value. (**c**) Effect of NEO on biofilm biomass formed on silicone sheets. Mean of dry weight ± SD of three independent sets of experiments is depicted on the y-axis, and * depicts *p* < 0.05. (**d**) Effect of NEO on cell adherence using buccal epithelial cells.

The impact of NEO on *C. auris* biofilm development was investigated by crystal violet staining, indicating that the levels of biofilm formed in NEO-treated samples significantly decreased in comparison with the untreated control (Figure 6a). The MTT assay showed a significant reduction in the metabolic activity against NEO-treated samples compared to non-treated samples (Figure 6b), with an approximate 70.6% decrease in the biofilm biomass with the presence of NEO relative to the non-treated ones (Figure 6c). Moreover, adherence of *C. auris* to buccal epithelial cells was determined to study host–pathogen interactions. Adherence of the fungus was reduced by NEO treatment, and fungal cells appeared visibly smaller and less dense on epithelial surfaces in treated cells compared with untreated controls (Figure 6d).

### 2.7. NEO Displays Negligible Haemolytic Activity

**Figure 7:**
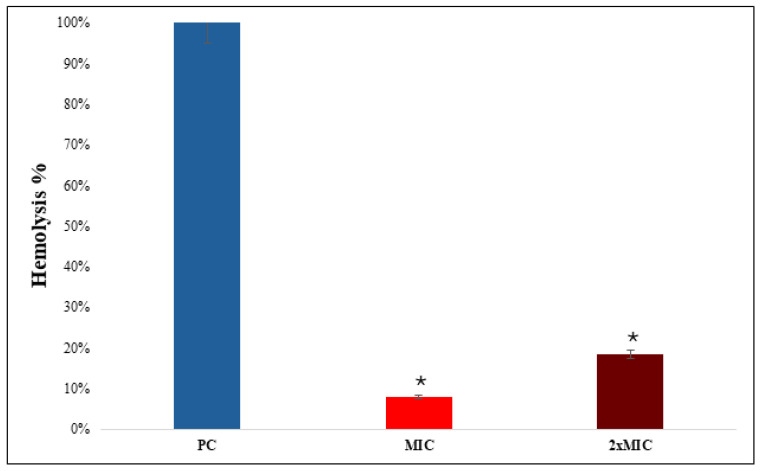
Hemolytic activity of NEO. The hemolytic activity is depicted as bar graph. Triton X-100 was used as a control. Means of O.D_570_ nm ± SD of three independent sets of experiments are depicted on Y-axis, and * depicts *p* < 0.05.

A hemolytic assay was performed using erythrocytes to assess the cytotoxicity of NEO against erythrocytes. The positive control of the assay, 1.0% *v*/*v* Triton X-100, showed complete hemolysis (98.5 ± 1.2%, *p* < 0.001) (Figure 7). On the other hand, NEO exhibited a low level of hemolytic activity (7.8 ± 0.9% at MIC and 15.6 ± 1.4% at 2× MIC), which was significantly less compared to the positive control (*p* < 0.01).

### 2.8. Chemical Analysis of Nutmeg Essential Oil

To identify the potential active constituents of NEO, gas chromatography was used. Based on the GC-MS analysis, the identified components are shown in Table 1; a total of 22 peaks were found and integrated with almost 100% TIC (2,885,455 a.u.), demonstrating high volatile purity without significant unresolved compounds or non-volatile impurities. The major constituents were Sabinene (27.30%), α-Pinene (14.51%), β-Pinene (13.47%), and D-Limonene (9.63%) and Terpinen-4-ol (9.41%).

## 3. Discussion

*C. auris*, an emerging multidrug-resistant fungal pathogen, represents an important public health threat worldwide because of its resistance to multiple antifungal drug classes and its persistence in clinical environments [9,25]. Recent evidence indicates that chemically characterized essential oils possess potent antifungal activity against a range of fungal pathogens and may serve as promising natural therapeutic agents [26,27]. Only a few studies have investigated the anti-*Candida* effects of essential oil constituents, which are reported to act through multiple mechanisms, including disruption of cell-membrane permeability, inhibition of ergosterol synthesis, induction of ROS, ion transport inhibition, and protein function impairment leading to fungal cell death [28,29]. In this study, we found broad-spectrum activity of NEO against *C. auris*, affecting multiple cellular targets, including cell wall integrity, membrane permeability, efflux pump function, oxidative stress, and biofilms, while displaying minimal hemolytic activity. Collectively, these findings support the potential of NEO as a natural antifungal agent and provide mechanistic insights into its mode of action. Although the MIC and MFC values of NEO against *C. auris* (500 and 650 µg/mL, respectively) were higher than those of conventional antifungal drugs, such concentrations are typical for essential oils due to their hydrophobic nature and complex multicomponent composition. Previous studies have reported MIC values ranging from 100 to 1000 µg/mL for *M. fragrans* and other aromatic plants against *Candida* species, including azole-resistant strains [17,24,30,31].

The cell wall of *C. auris* is an essential barrier that supports the integrity, shape, and virulence of the cell with its major constituents (chitin and β-1,3-glucan) [32]. Disruption of these components often triggers compensatory remodeling responses. In this study, NEO treatment resulted in pronounced alteration in cell wall polysaccharide exposure, suggesting interference with normal cell wall components and organization [33]. These findings are consistent with those reported for other essential oils, which act against fungal cell walls directly by disrupting the membrane. Cinnamon bark essential oil induces cell wall remodeling in *C. albicans* [34], while clove and lemongrass oils compromises the envelope integrity among several *Candida* species including *C. auris* [35,36,37]. Although membrane permeabilization and ergosterol binding are considered the primary mode of action for several essential oils, secondary effects on enzymes involved in cell wall synthesis such as β-1,3-glucan synthase and chitin synthase have also been proposed for compounds like cinnamaldehyde and eugenol [38]. In general, these findings highlight the antifungal action of NEO disruption of the cell wall and may contribute to its efficacy in resistant pathogens. Further molecular studies are required to clarify the precise targets of NEO, including potential effects on genes such as FKS1 (encoding β-glucan synthase) and chitin synthase genes. In fungi like *C. auris*, ergosterol plays a critical role in the structure of plasma membrane and controls membrane fluidity, permeability, and integrity [39]. Changes in ergosterol biosynthesis represent a well-defined mode of action for antifungals and a major resistance in pathogenic *Candida* species. In the present study, NEO treatment compromised membrane integrity, as evidenced by hypersensitivity to SDS, increased PI uptake, leading to increased cell permeability. In addition, the quantitative ergosterol determination showed a significant decrease in the ergosterol level, indicating direct influence on the biosynthesis of ergosterol. This result is similar with cinnamon and clove essential oils, reducing ergosterol levels and disrupting membrane stability in *Candida* species [36,40,41]. While the mode of action of essential oils is mainly due to perturbation of membranes through hydrophobic interactions and ergosterol binding, their constituents may indirectly interfere with enzymes in the ergosterol pathway, like azoles, which target 14α-demethylase (CYP51) in lanosterol [37]. The NEO-induced effect on *C. auris* probably results from multi-targeted disruption, including ergosterol depletion and subsequent permeability changes, leading to the increased susceptibility of resistant strains to NEO treatment, but the molecular-level target is not studied in this study. ABC and MFS efflux pumps are two of the primary efflux pumps that play critical roles in antifungal drug resistance in *C. auris*, particularly to azoles [42]. Accordingly, the efflux-associated observation in this work is most likely explained by a membrane-linked disequilibrium of transporter function to support membrane-disruptive antifungal action of NEO, and the present results did not provide mechanistic proof for a direct inhibition of drug efflux pumps but suggest reduced functionality. Subsequent molecular analyses, such as transporter expression and membrane biophysical assays, would be necessary to dissect the specific mechanisms of efflux impairment.

Oxidative stress is also a major mediator of antifungal-stimulated cytotoxicity in yeast. It results from excessive cellular ROS accumulation that disrupts the electrochemical gradient and stimulates programmed cell death [43]. Treatment of *C. auris* with NEO induced a significant enhancement in the intracellular ROS levels, comparable to exposure to hydrogen peroxide. A reduced extent of ROS accumulation and subsequent growth rescue upon combined treatment of AA further supports the role of ROS in NEO-induced cytotoxicity. Comparable ROS-related effects have been described for *Candida* spp. challenged with oxidative stress-generating antifungals, reinforcing the functional role of ROS in fungal growth inhibition [17,39].

High concentrations of ROS are responsible for lipid peroxidation that brings instability to membranes and reduces cellular integrity. Accordingly, increased TBARS levels after NEO treatment represent increased lipid peroxidation that was partially inhibited by antioxidant therapy. These results concur with earlier works revealing the role of EOs’ constituents in mediating oxidative injury to the membrane of *Candida* spp., resulting in antifungal effects [44,45,46]. Besides damage to the cell membranes, oxidative stress generated by the NEO could also increase nuclear damage, as shown by increased DAPI fluorescence in NEO-treated cells, indicative of DNA fragmentation. DNA damage mediated by ROS is generally recognized to play a prominent role in essential oil-mediated antifungal activity and apoptotic cell death in yeast models [39,44,45]. Overall, these data indicate that NEO has an inhibitory effect against fungi through ROS-induced cellular damage by synergistic disruption of the membrane lipids and nuclear integrity. The ROS determinations and antioxidant recovery in the experiments are indirect; therefore, these results represent a functional metric of oxidative stress involvement rather than a confirmed cell death mechanism. However, the combined evidence of ROS accumulation, lipid peroxidation, and nuclear damage emphasize oxidative stress as one of the important factors contributing to NEO’s multifunctional antifungal activity against *C. auris*.

Biofilm production and the ability to adhere to host surfaces are important virulence factors of *C. auris* that allow for colonization on both biotic and abiotic substrates, escape from immune system defenses, and resistance to antifungal therapy [7,47]. In the current study, NEO displayed a significant reduction in biofilm biomass and metabolic activity, as measured by crystal violet and MTT assays, respectively, revealing efficient inhibition towards both biofilm formation and cellular viability in the biofilm matrix. Besides biofilm inhibition, NEO also significantly decreased adherence of *C. auris* to buccal epithelial cells, indicating an obstruction in early host–pathogen association. Adherence to *Candida* species is predominantly mediated by the cell wall-associated adhesins and surface hydrophobicity, which are both affected by changes in the cell wall structure and membrane permeability [26]. Essential oils disturb the fungal cell envelope, which may in turn disrupt adhesin function and eventually decrease ex vivo epithelial adherence with a corresponding loss of virulence [47,48]. Accordingly, the combined anti-biofilm and anti-adherence activities found in this study indicate that NEO can potentially attenuate *C. auris* virulence by interfering with more than one virulence-associated mechanism rather than primarily inhibiting planktonic growth. Regarding the host compatibility, hemolysis tests showed less cytotoxicity. But alone, it is insufficient to establish overall cytotoxic safety, as erythrocytes lack metabolic and signaling pathways that are present in other mammalian cell types [49]. Safety-related conclusions are therefore confined to early biocompatibility in the current study, and subsequent evaluation with mammalian cell types and animal models would be necessary prior to confirming any therapeutic potential.

Taken together, the capacity of NEO to prevent biofilm formation and host cell adhesion, along with its low hemolytic activity, emphasizes its potential as a candidate for use in topical, surface-associated applications, like how it can be incorporated into biopolymers and other wound-dressing materials. However, detailed cytotoxicity profiling is still an essential need in order to translate these findings into clinical relevance.

## 4. Materials and Methods

### 4.1. Materials

Chemicals and reagents used in the present study were obtained from known companies. Yeast Extract Peptone Dextrose (YPD) broth, agar, rhodamine 6G (R6G), Nile red (NR), 2,4-dinitrophenol (DNP), 2-deoxy-D-glucose (2-DOG), dimethyl sulfoxide (DMSO), potassium hydroxide (KOH), and n-heptane were procured from HiMedia Laboratories Mumbai, India. D-glucose, sodium chloride (NaCl), and potassium chloride (KCl) were purchased from Thermo Fisher Scientific (Waltham, MA, USA). Crystal violet (CV) and Calcofluor White (CFW) were obtained from Sigma-Aldrich (St. Louis, MO, USA). The nutmeg essential oil was obtained from a commercial source, and a single batch was used during the entire study.

### 4.2. Culture Conditions and Microbial Strain

The *C. auris* reference strain CBS10913T was employed in all the experiments during this study. The organism was grown in YEPD broth (*w*/*v*, 1% yeast extract, 2% peptone, and 2% dextrose. Agar in 2% (*w*/*v*) was included for solid medium. The strain was inoculated on YEPD agar plates or liquid medium, depending on the specific requirement prior to experiments.

### 4.3. Antifungal Activity of NEO Against C. auris

To check the antifungal activity of NEO against *C. auris*, we conducted microbroth dilution and spot assays.

### 4.4. Microbroth Dilution and Spot Assays

The MIC of NEO against *C. auris* was determined using the microbroth dilution assay, as previously described [42]. Phenotypic susceptibility of *C. auris* to NEO was evaluated by spot dilution assays using a modified drop dilution method, following previously described methods [50]. Briefly, *C. auris* cells were resuspended in sterile normal saline to an optical density of 0.1 at 600 nm, corresponding to approximately 1 × 10^6^ CFU/mL. Fivefold serial dilutions of the suspension were spotted (6 µL) onto YEPD agar plates ± NEO. Plates were incubated at 30 °C for 48 h and growth inhibition was assessed by visual observation of colony formation.

### 4.5. Static and Cidal Assessment

For the assessment of the static and cidal activity of NEO, *C. auris* cells (OD_600_ = 0.1) were inoculated into test tubes containing YEPD medium with and without NEO, which was incubated at 30 °C. After incubation, OD_600_ was recorded using a spectrophotometer, following which 100 µL of inoculum was taken out and re-inoculated in YEPD media without NEO, and the next day, OD_600_ was measured again [39].

### 4.6. Assessment of Cell Viability

The cell viability assay of *C. auris* exposed to NEO was performed by using trypan blue exclusion, as previously described [39]. This technique is based on the selective uptake of trypan blue by cells with damaged membranes, allowing viable and non-viable cells to be distinguished. Overnight-grown *C. auris* cells in YEPD were resuspended in sterile PBS to an OD_600_ of 0.1. Then, cells were inoculated with NEO at sub-inhibitory, inhibitory, and fungicidal concentrations, along with an untreated control, and incubated at 30 °C for 24 h. After incubation, cells were collected by centrifugation and washed twice with PBS and then resuspended in PBS. The cell suspension was mixed at a 1:1 with 0.4% trypan blue solution and incubated at 30 °C for 15 min. Cells were counted with a hemocytometer using an optical microscope (Nikon Eclipse, Tokyo, Japan). Cell viability was expressed as a percentage of viable cells using the following formula:

% Viability = [Number of Unstained Cells/(Number of Unstained + Stained Cells)] × 100.(1)

### 4.7. Fluorescence-Based Analysis of Cell Wall Constituents

To assess the impact of NEO on the cell wall composition of *C. auris*, chitin and β-1,3-glucan content was analyzed via fluorescence microscopy. Exponentially growing *C. auris* cells (2.5 × 10^6^ cells/mL in YEPD) were harvested, washed, and treated with NEO, with untreated cells as the control. After treatment, the cells were fixed with the help of 4% (*v*/*v*) paraformaldehyde in PBS for 30 min at room temperature and washed twice with PBS to remove residual fixative. For selective staining, fixed cells were incubated with 5 µg/mL CFW to visualize chitin and 100 µg/mL Aniline Blue to visualize β-1,3-glucan, each for 30 min in the dark at room temperature. After staining, samples were imaged using a Nikon Eclipse fluorescence microscope (100× oil-immersion objective) and analyzed using Nikon NIS-Elements software (version 4.6) [51].

### 4.8. Evaluation of Ergosterol Levels

To determine the ergosterol content in *C. auris*, the alcoholic KOH extraction method was used, as previously described [52]. The cells were treated with NEO and incubated for 4 h at 30 °C, while untreated cells served as the control. After incubation, the cells were collected, washed with PBS, and resuspended in 3 mL of 25% (*w*/*v*) alcoholic KOH (ethanol–water, 3:2). Sterols were extracted by heating the suspension at 85 °C for 1 h, followed by extraction with 5 mL of petroleum ether. The organic phase was dried under a stream of nitrogen, and the residue was dissolved in 1 mL of petroleum ether for analysis. Absorbance was measured at 230 and 281.5 nm with UV–visible spectrophotometer (Genetix-GMB-580, Biotech Asia Pvt Ltd., Bengaluru, India). Ergosterol content was expressed as a percentage of the wet cell pellet weight according to the following formula:

% Ergosterol = [% Ergosterol + % 24(28)-DHE] − % 24(28)-DHE(2)

where A281.5 and A230 are absorbances at 281.5 nm and 230 nm, respectively; 290 and 518 are the molar extinction coefficients (% per cm) for crystalline ergosterol and 24(28)-dehydroergosterol (DHE), respectively; and F is the dilution factor in petroleum ether.

### 4.9. Rhodamine 6G Extracellular Efflux Assay

The Rhodamine 6G (R6G) efflux assay was performed, as described previously [50]. Exponentially growing *C. auris* cells were treated with NEO and incubated for 5 h at 30 °C, alongside untreated controls. After incubation, the cells were harvested, washed twice with PBS, and reconstituted to a 2% (*w*/*v*) cell suspension (approximately 1 × 10^8^ cells/mL) in glucose-free PBS. To inhibit cellular metabolism, the cells were de-energized by incubation in glucose-free PBS containing 5 mM 2-deoxy-D-glucose (2-DOG) and 5 mM 2,4-dinitrophenol (DNP) for 45 min at 30 °C. R6G was then added to a final concentration of 10 µM, and cells were incubated for 40 min at 30 °C to allow dye uptake. Following equilibration, the cells were washed and resuspended in glucose-free PBS to a 2% (*w*/*v*) suspension. For efflux measurements, 1 mL samples were collected at specified time points and centrifuged (9000× *g*, 2 min), and the absorbance was measured at 527 nm using a UV–visible spectrophotometer (Genetix-GMB-580, Biotech Asia Pvt Ltd., Bengaluru, India).

### 4.10. Rhodamine 6G and Nile Red Accumulation Assay

Intracellular accumulation of R6G and NR in *C. auris* in response to NEO was investigated using fluorescence-based accumulation assays, as previously described [53]. *C. auris* cells were grown overnight at 30 °C and then incubated for an additional 2 h at 30 °C in the presence of 200 µg/mL NEO, with untreated cells serving as controls. The cells were harvested, washed twice with ice-cold 10 mM PBS, and resuspended in 1.0 mL PBS supplemented with 2% (*w*/*v*) glucose. The cell suspensions were then incubated for 30 min at 30 °C in the presence of either R6G (4 µM) or NR (7 µM) fluorescent dye. Following incubation, the cells were centrifuged, washed twice with ice-cold PBS to remove unbound dye, and resuspended in PBS. The final cell pellet was analyzed using a fluorescence microscope (Nikon Eclipse, Tokyo, Japan) at 100× magnification.

### 4.11. ROS and DNA Damage

Reactive oxygen species generation and DNA damage in *C. auris* in the presence of NEO were assessed by using DCFDA and DAPI staining assays, followed by fluorescence microscopy. *C. auris* cells were treated with NEO and incubated for 3 h at 30 °C with shaking, while untreated cells served as the control. At the end of treatment, cells were washed with PBS to remove residual medium and stained with 10 µM DCFDA and 1 µg/mL DAPI in PBS. The stained cells were observed under a fluorescence microscope (Nikon Eclipse, Tokyo, Japan) at 100× magnification, and images were acquired using NIS-Elements software (Nikon, Tokyo, Japan; version 4.6). Fluorescence intensity was used to semi-quantitatively assess ROS generation and nuclear DNA damage [34,40].

### 4.12. Lipid Peroxidation and Oxidative Stress in C. auris Using TBARS Assay

Lipid peroxidation in *C. auris* after treatment with NEO was assessed using a thiobarbituric acid reactive substance (TBARS) assay, according to previously published protocols with slight modifications [40,54]. Briefly, cells were exposed to NEO for 4 h at 30 °C, with untreated cells serving as negative controls and H_2_O_2_-treated cells as positive controls. Following treatment, the cells were lysed, and TBARS levels were measured to assess malondialdehyde (MDA) production as an index of lipid peroxidation. The absorbance of the reaction mixture was measured at 535 nm using a UV-vis spectrophotometer (Genetix GMB-580, Biotech Asia Pvt. Ltd., Bengaluru, India). Lipid peroxidation levels were expressed as MDA equivalent (nmol/mL) compared with a standard calibration curve.

### 4.13. Quantification of Biofilm Metabolic Activity

The effect of NEO on the metabolic activity of *C. auris* biofilms was investigated using a 3-(4,5-dimethylthiazol-2-yl)-2,5-diphenyltetrazolium bromide (MTT) assay, according to previously described methods [55]. Biofilms were established in 96-well microtiter plates containing exponential phase *C. auris* cells and diluted to 1 × 10^6^ cells/mL in YEPD. Cell suspensions (100 µL/well) were dispensed into the wells and treated with NEO, with untreated wells serving as controls. The plates were incubated at 37 °C for 24 h to allow biofilm formation. Subsequently, 50 µL of MTT solution (stock: 5 mg/mL, diluted by 1:5 in prewarmed 0.15 M PBS) was added into each well and incubated for 5 h at 37 °C to allow reduction of the MTT to formazan by metabolically active cells. The resulting formazan was dissolved by the addition of 200 µL DMSO to each well, and absorbance was measured at 600 nm on a UV–visible 96-well plate reader.

### 4.14. Quantification of Biofilm Biomass

To measure the impact of NEO on *C. auris* biofilm biomass, a dry weight assay was performed using silicone squares, as previously described [42]. Sterile PEGDM-SRSG squares (1.5 × 1.5 cm) were pretreated by overnight incubation at 37 °C in bovine serum (Sigma-Aldrich, St. Louis, MO, USA) to promote cell adhesion, followed by two washes with PBS to remove residual serum. *C. auris* cells were resuspended at an OD_600_ of 0.2 (approximately 2 × 10^6^ cells/mL) in Spider medium. Two milliliters of this suspension were pipetted into each well of a sterile 12-well microtiter plate containing preweighed silicone squares. The plates were incubated at 37 °C for 90 min with gentle shaking to promote initial adhesion. The squares were then washed with 2 mL PBS to remove non-adherent cells and transferred to a new 12-well plate containing Spider medium with NEO (wells without NEO served as controls). Biofilm formation was allowed to continue for 60 h at 37 °C, after which silicone squares were washed twice in PBS to remove nonadherent cells, air-dried at room temperature for 48 h, and weighed. Biofilm biomass was calculated by subtracting the preweighed dry mass of the silicone square from its weight after biofilm formation (mg).

### 4.15. Adherence to Buccal Epithelial Cells

To evaluate the influence of NEO on *C. auris* adherence, an in vitro-mediated adhesion assay was performed as previously described [54]. Buccal epithelial cells were collected from one of the co-authors, after obtaining informed consent. This non-invasive procedure involved gentle scraping on the buccal mucosa of cheeks using sterile cotton swabs and posed no risk to any participant. *C. auris* cells were collected and suspended in 2 mL Spider medium at a concentration of 1 × 10^6^ cells/mL. Epithelial cells were then detached by gentle shaking in PBS. For the adherence assay, 1 mL of the *C. auris* suspension was mixed with 1 mL of the epithelial cell suspension in sterile test tubes. NEO was used at 200 µg/mL for treated samples, and those without NEO served as controls. The mixtures were incubated at 37 °C for 2 h to allow cell–cell interaction. After incubation, 50 µL of 0.4% (*w*/*v*) trypan blue was added to each tube, followed by gently vortexing to ensure uniform staining. The stained suspension (10 µL) was loaded onto a Neubauer hemacytometer and examined under a light microscope at 40× magnification (Nikon Eclipse, Tokyo, Japan). Adherent *C. auris* cells were quantified by counting the number of yeast cells that were attached to epithelial cells.

### 4.16. Haemolytic Activity

The hemolytic activity of NEO on RBCs was assessed as previously described [55].

RBCs were collected from a healthy donor (one of the co-authors) after obtaining informed consent. The cells were washed three times by centrifugation (2000 rpm, 2 min) with sterile PBS to remove plasma and debris and then resuspended to a final concentration of 4% (*v*/*v*) in PBS. NEO was added to 1 mL of the RBC suspension at MIC and 2× MIC to obtain a final volume of 1 mL. The samples were incubated at 37 °C for 35 min with gentle agitation. After incubation, the samples were centrifuged, and the supernatant was collected to measure hemoglobin release at 540 nm using a UV–visible spectrophotometer. Negative controls consisted of RBCs suspended in PBS alone (A_blank_), while positive controls used hRBCs in 1% (*v*/*v*) Triton X-100 (A_Triton_) to induce complete hemolysis. The percentage of hemolysis was calculated using the equation:

% Hemolysis = [(A_test sample_ − A_blank_)/(A_Triton_ − A_blank_)] × 100 (3)

where A_test_ sample, A_blank_, and A_Triton_ represent absorbances of the test sample, negative control, and positive control, respectively.

### 4.17. GC-MS Characterization of NEO

A sample of NEO used in the present work was analyzed for its chemical composition by GC-MS. Compound characterization was performed through the comparison between mass spectra and those available in the NIST 23 MS library, using literature retention indices. The data were calculated as a proportion of the total area for each detected compound by relative peak area without using an internal standard.

### 4.18. Statistical Analysis

Each experiment was carried out in triplicate (*n* = 3). Student’s *t*-test was used to test for statistical differences between control and treatment groups. Results are given as the mean ± SD, and *p* < 0.05 was considered significant.

## 5. Conclusions

The present study shows that NEO is a complex mixture of various components that exerts distinct antifungal activity against *C. auris* through multiple interrelated cellular effects. Against the standardized reference strain, NEO demonstrated fungicidal activity with marked effects on fungal cell wall and membrane integrity, including ergosterol depletion and increase in membrane permeability and inhibition of efflux transporter activity. These membrane-related effects were correlated with increased intracellular oxidative stress, leading to lipid peroxidation and nuclear alterations, indicating a multifactorial positioning of antifungal action. NEO also suppressed biofilm formation and host colonization; both parameters represent important factors in *C. auris* persistence and pathogenesis. The important thing is that the documented biological activities can be attributed to all these major components and their possible synergistic effect but not to a specific compound and that all mechanisms are considered only functional and phenomenological. Preliminary biocompatibility assessment was evaluated through hemolytic assay, and it revealed the low level of erythrocyte damage, but alone it cannot be used as proof of complete safety. Mammalian cell lines and in vivo models need to be employed for full safety profiling before clinical applications. In addition, the quite high MIC and MFC results found further suggest that the activity of essential oils for systemic use is pharmacodynamically limited due to solubility restrictions and dose-related toxicity. In view of this, the antifungal application of NEO is most suited to topical, localized, or surface use rather than being a single systemic treatment. Future research can include (i) molecular-level analysis such as transcriptomic and proteomic approaches to better understand stress-response and membrane-related targeted pathways affected by NEO, (ii) evaluations of in vivo antifungal activity in appropriate animal infection models for determination of both efficacy and safety, (iii) formulation methodologies like nanoparticles, hydrogels, and biopolymer-based drug delivery towards improved stability; availability at site of action assumes obligation. Furthermore, broader studies that include diverse clinical isolates and MDR fungi will be necessary to determine the application of NEO for a large spectrum of fungi. In summary, this study identifies nutmeg essential oil as a molecularly multi-target natural antifungal with potential topical and adjunctive use against *C. auris*. The effective balance of chemical characterization, pharmacological realism, and advanced formulation strategies will be an important parameter to drive NEO from in vitro findings to meaningful biomedical implications.

## 6. Limitations of Current Study

This study has a few limitations that should be mentioned. First, the composition of NEO was characterized by GC-MS, and all assays were performed using a single commercial batch. The chemo-profile was consistent with the reported literature, but batch-to-batch variation was not evaluated. Secondly, the relatively high MIC and MFC values obtained, typical of complex essential oil mixtures, restrict the possibility for systemic therapeutical application. Thirdly, the inferences concerning the role of efflux pumps are from functional fluorescence assays that can only give indirect evidence and cannot differentiate between direct transporter effects and membrane-dependent impairment. Thus, kinetics should be viewed as phenomenological and not mechanistic. Fourthly, it is the erythrocyte hemolysis that was examined as an indication of a preliminary biocompatibility indicator and not as proof of complete safety. No mammalian cell line or in vivo toxicity assays were carried out, limiting safety conclusions. Lastly, no molecular-level analyses of gene or protein regulation were performed, and antifungal activity was only evaluated against a reference *C. auris* strain, raising the need for validation across clinical isolates.

## Figures and Tables

**Table 1:** Major constituents of NEO analyzed by GC-MS.

Compound	Area %	Similarity Index	CAS
Sabinene	27.30	95	3387-41-5
α-Pinene	14.51	93	80-56-8
β-Pinene (isomer)	13.47	95	18172-67-3
D-Limonene	9.63	91	5989-27-5
Terpinen-4-ol	9.41	90	562-74-3
γ-Terpinene	3.41	89	99-85-4
β-Myrcene	2.56	86	123-35-3
p-Cymene	2.10	87	99-87-6
trans-Isomyristicin	2.32	78	18312-21-5
Isoelemicin	1.48	72	487-12-7

## Data Availability

The original contributions presented in this study are included in the article/Supplementary Material. Further inquiries can be directed to the corresponding authors.

## Supplementary Materials

The following supporting information can be downloaded at: https://www.mdpi.com/article/10.3390/ph19020233/s1, The chromatograms and corresponding mass spectral data obtained from GC-MS used for compound identification can be found in Supplementary File S1.
