# Supplementary material for: Unmasking the Fungicidal Potency and Multifaceted Mechanisms of Nutmeg Essential Oil Against Candida auris
Source: Pharmaceuticals (Basel). 2026 Jan 29;19(2):233. doi: 10.3390/ph19020233 (PMC12943223; doi:10.3390/ph19020233)

Sample Information

Analyzed by : Admin
Analyzed : 12/29/2025 11:04:17 AM
Sample Type : Unknown
Level # : 1
Sample Name : AKRI_01
Sample ID :
IS Amount : [1]=1
Sample Amount : 1
Dilution Factor : 1
Vial # : 1
Injection Volume : 1.00
Data File : D:\Amity\Data file\dec 2025\AKRI01_291225.qgd
Org Data File : D:\Amity\Data file\dec 2025\AKRI01_291225.qgd
Method File : D:\Amity\Method\dec 2025\AKRI01_291225.qgm
Org Method File : D:\Amity\Method\dec 2025\AKRI01_291225.qgm
Report File :
Tuning File : C:\GCMSsolution\System\Tune1\AUTOTUNE DEC 25.qgt
Modified by : Admin
Modified : 12/29/2025 11:53:17 AM

GC Chromatogram AKRI_01

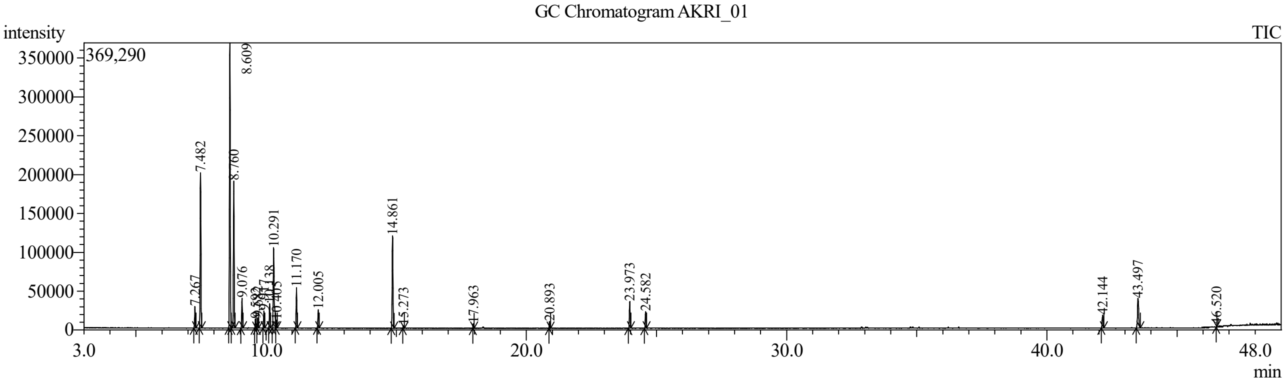

Peak Report TIC

Peak#	R.Time	Area	Area%	A/H	imilarity	Name
1	7.267	51297	1.78	1.84	84	Bicyclo[3.1.0]hex-2-ene, 2-methyl-5-(1-met
2	7.482	418818	14.51	2.10	93	.alpha.-Pinene
3	8.609	787593	27.30	2.15	95	Bicyclo[3.1.0]hexane, 4-methylene-1-(1-me
4	8.760	388682	13.47	2.06	95	Bicyclo[3.1.1]heptane, 6,6-dimethyl-2-met
5	9.076	73934	2.56	1.93	86	.beta.-Myrcene
6	9.592	21151	0.73	1.83	81	Bicyclo[2.2.1]hept-2-ene, 2-[(2,4-dinitroph
7	9.682	24750	0.86	1.84	81	.alpha.-Phellandrene
8	9.917	42104	1.46	1.93	79	Cyclohexene, 3-methyl-6-(1-methylethylid
9	10.138	60672	2.10	1.94	87	p-Cymene
10	10.291	278007	9.63	2.69	91	D-Limonene
11	10.405	14130	0.49	1.36	71	3-Bromothiophenol, S-(2-methylpropionyl
12	11.170	98304	3.41	1.88	89	.gamma.-Terpinene
13	12.005	45504	1.58	1.94	79	(+)-4-Carene
14	14.861	271634	9.41	2.29	90	Terpinen-4-ol
15	15.273	5638	0.20	1.73	77	3-Ethoxy-2-bromo-1-propanol
16	17.963	8818	0.31	1.55	81	1,2,4-Oxadiazol-3(2H)-one, 5-phenyl-
17	20.893	15391	0.53	1.78	66	Benzylphenethylamine, N-methoxycarbon
18	23.973	66894	2.32	1.95	78	trans-Isomyristicin
19	24.582	42780	1.48	2.01	72	Isoelemicin
20	42.144	41853	1.45	2.56	79	4H-1,2,4-triazol-3-ol, 5-[(phenylmethyl)thi
21	43.497	125896	4.36	3.33	88	Triphenylphosphine oxide
22	46.520	1605	0.06	0.75	87	Propanoic acid, anhydride
		2885455	100.00			

Library

<< Target >>
Line#:1 R.Time:7.265(Scan#:854) MassPeaks:7
RawMode:Averaged 7.260-7.270(853-855) BasePeak:93.10(9899)
BG Mode:Calc. from Peak Group 1 - Event 1 Scan

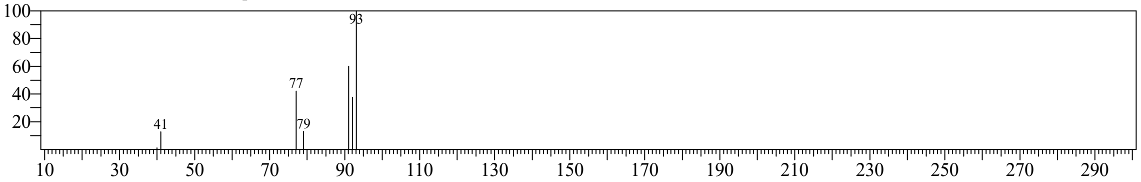

Hit#:1 Entry:8410 Library:NIST23s.lib
SI:84 Formula:C10H16 CAS:2867-05-2 MolWeight:136 RetIndex:939
CompName:Bicyclo[3.1.0]hex-2-ene, 2-methyl-5-(1-methylethyl)- $$ 3-Thujene $$ .alpha.-Thujene $$ Thujene, .alpha.- $$ Origanene $$ 5-Isopropyl-2-met

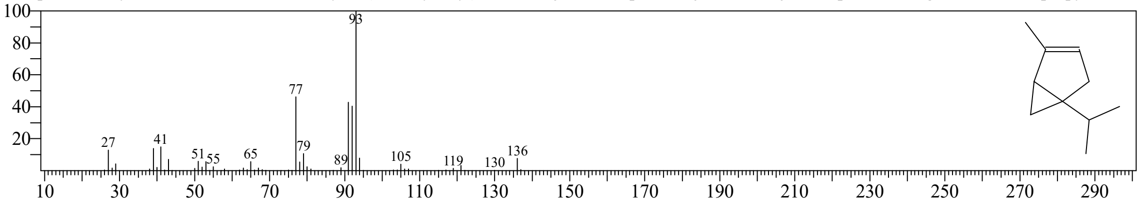

Hit#:2 Entry:8437 Library:NIST23s.lib
SI:84 Formula:C10H16 CAS:99-83-2 MolWeight:136 RetIndex:1017
CompName:.alpha.-Phellandrene $$ 1,3-Cyclohexadiene, 2-methyl-5-(1-methylethyl)- $$ .alpha.-Fellandrene $$ p-Mentha-1,5-diene $$ 5-Isopropyl-2-methy

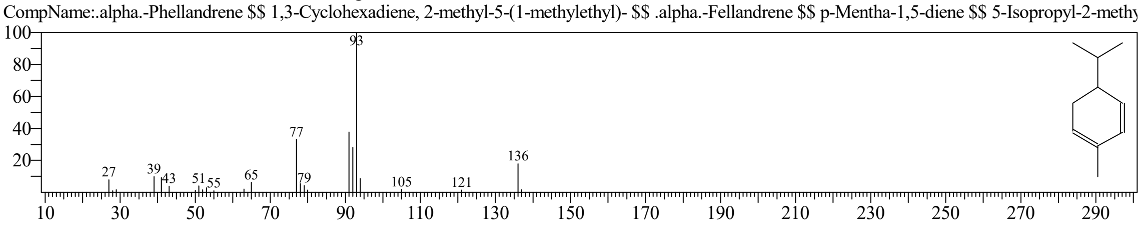

Hit#:3 Entry:11134 Library:NIST23-1.lib
SI:82 Formula:C8H12N2 CAS:109746-10-3 MolWeight:136 RetIndex:1191
CompName:1,4-Methano-1H-Cyclopropa[d]pyridazine, 4,4a,5,5a-tetrahydro-6,6-dimethyl-, (1.alpha.,4.alpha.,4a.alpha.,5a.alpha.)-

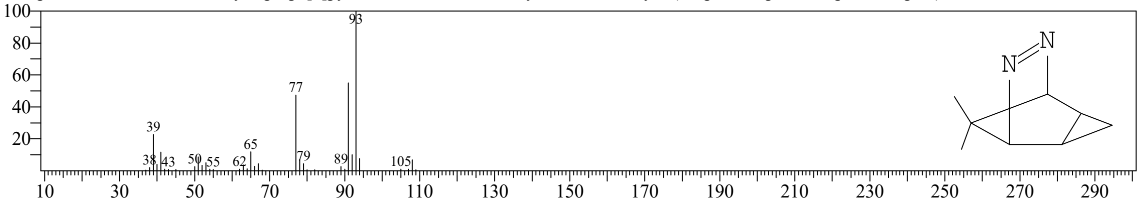

Hit#:4 Entry:11390 Library:NIST23-1.lib
SI:82 Formula:C10H16 CAS:28634-89-1 MolWeight:136 RetIndex:924
CompName:Bicyclo[3.1.0]hex-2-ene, 4-methyl-1-(1-methylethyl)- $$ 2-Thujene $$ .beta.-Thujene $$ 1-Isopropyl-4-methylbicyclo[3.1.0]hex-2-ene $$

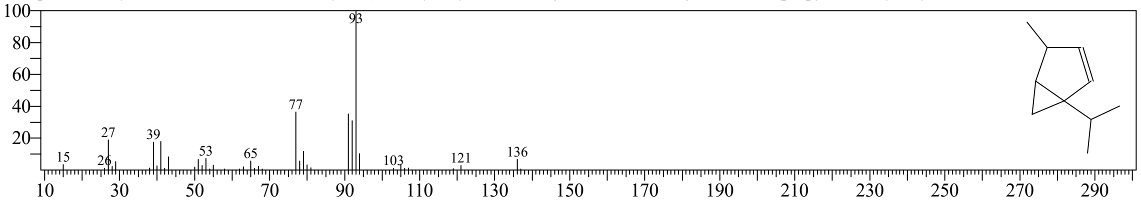

Hit#:5 Entry:172340 Library:NIST23-1.lib
SI:81 Formula:C13H12N2O4S CAS:0-00-0 MolWeight:292 RetIndex:2584
CompName:Bicyclo[2.2.1]hept-2-ene, 2-[(2,4-dinitrophenyl)thio]- $$ 2-[(2,4-dinitrophenyl)thio]bicyclo[2.2.1]hept-2-ene $$ bicyclo[2.2.1]hept-2-en-2-yl 2,

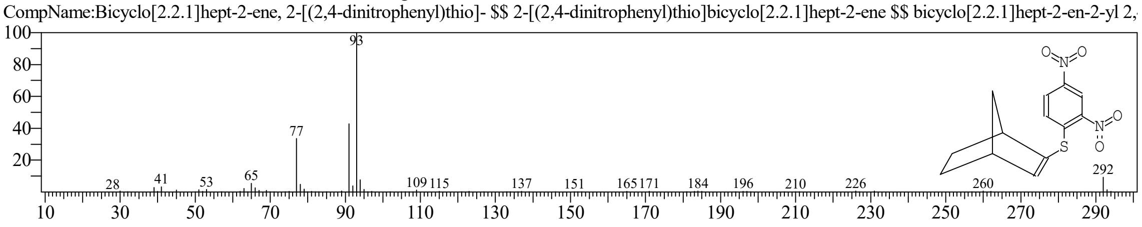

<< Target >>
Line#:2   R.Time:7.480(Scan#:897) MassPeaks:26
RawMode:Averaged 7.475-7.485(896-898) BasePeak:93.10(51909)
BG Mode:Calc. from Peak Group 1 - Event 1 Scan

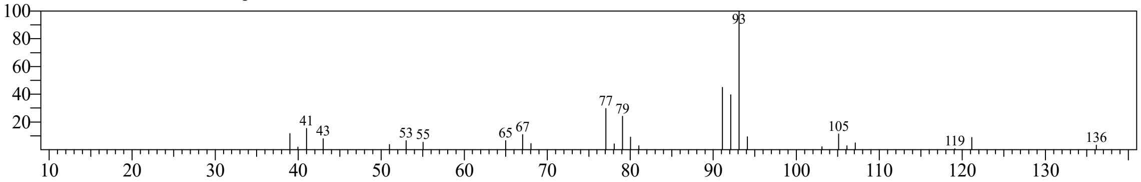

Hit#:1 Entry:8451 Library:NIST23s.lib
SI:93 Formula:C10H16 CAS:80-56-8 MolWeight:136 RetIndex:947
CompName:.alpha.-Pinene $$ Bicyclo[3.1.1]hept-2-ene, 2,6,6-trimethyl- $$ 2-Pinene $$ 2,6,6-Trimethylbicyclo[3.1.1]hept-2-ene $$ Pinene, .alpha. $$ 2,6,6

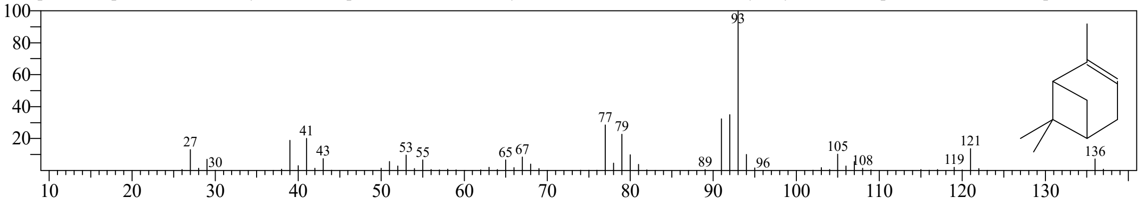

Hit#:2 Entry:11415 Library:NIST23-1.lib
SI:93 Formula:C10H16 CAS:7785-70-8 MolWeight:136 RetIndex:947
CompName:(1R)-2,6,6-Trimethylbicyclo[3.1.1]hept-2-ene $$ 1R-.alpha.-Pinene $$ Bicyclo[3.1.1]hept-2-ene, 2,6,6-trimethyl-, (1R)- $$ 2,6,6-Trimethylbicy

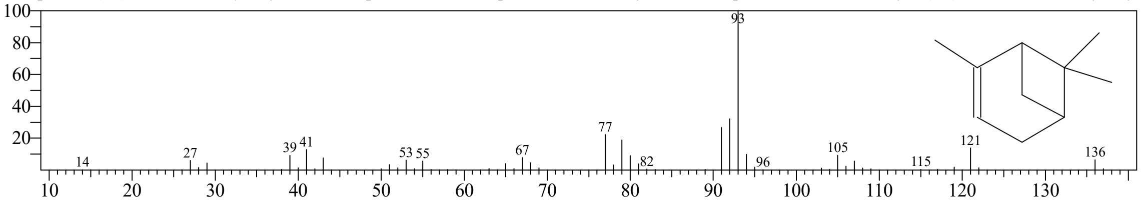

Hit#:3 Entry:11414 Library:NIST23-1.lib
SI:92 Formula:C10H16 CAS:7785-26-4 MolWeight:136 RetIndex:947
CompName:(1S)-2,6,6-Trimethylbicyclo[3.1.1]hept-2-ene $$ 1S-.alpha.-Pinene $$ (-)-.alpha.-Pinene $$ L-.alpha.-Pinene $$ Bicyclo[3.1.1]hept-2-ene, 2,6,6-

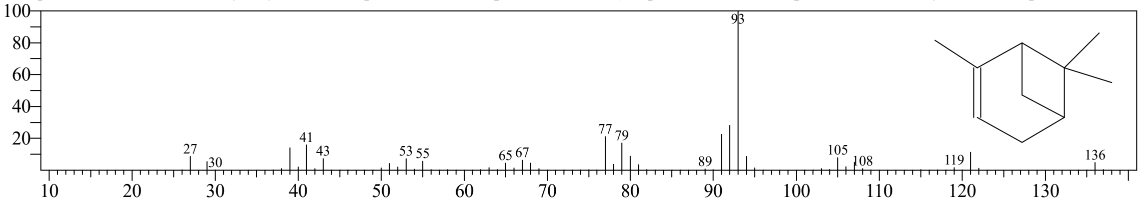

Hit#:4 Entry:11413 Library:NIST23-1.lib
SI:92 Formula:C10H16 CAS:4889-83-2 MolWeight:136 RetIndex:975
CompName:Bicyclo[3.1.1]hept-2-ene, 3,6,6-trimethyl- $$ 2-Norpinene, 3,6,6-trimethyl- $$ 3-Methyl-apopinene $$ 3,6,6-Trimethylbicyclo[3.1.1]hept-2-ene

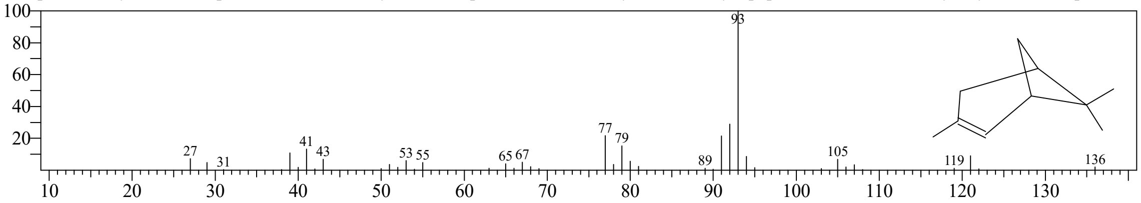

Hit#:5 Entry:8449 Library:NIST23s.lib
SI:92 Formula:C10H16 CAS:488-97-1 MolWeight:136 RetIndex:906
CompName:Tricyclo[2.2.1.0(2,6)]heptane, 1,3,3-trimethyl- $$ Cyclofenchene $$ Tricyclo[2.2.1.02,6]heptane, 1,3,3-trimethyl- $$

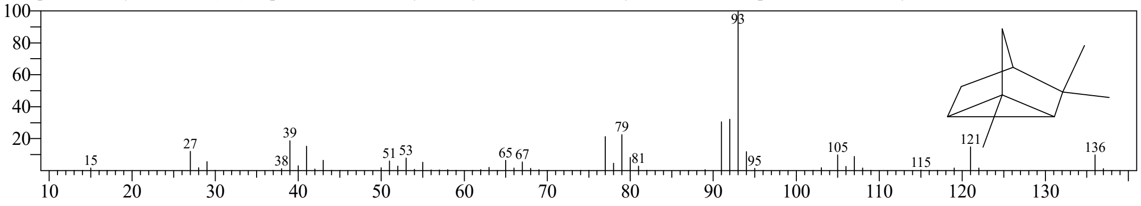

<< Target >>
Line#:3   R.Time:8.610(Scan#:1123) MassPeaks:30
RawMode:Averaged 8.605-8.615(1122-1124) BasePeak:93.10(96442)
BG Mode:Calc. from Peak Group 1 - Event 1 Scan

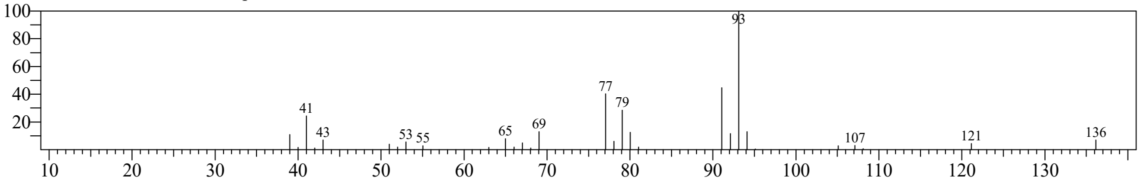

Hit#:1 Entry:11405 Library:NIST23-1.lib
SI:95 Formula:C10H16 CAS:3387-41-5 MolWeight:136 RetIndex:968
CompName:Bicyclo[3.1.0]hexane, 4-methylene-1-(1-methylethyl)- $$ 1-Isopropyl-4-methylenebicyclo[3.1.0]hexane $$ 4(10)-Thujene $$ Sabinen $$ Sabin

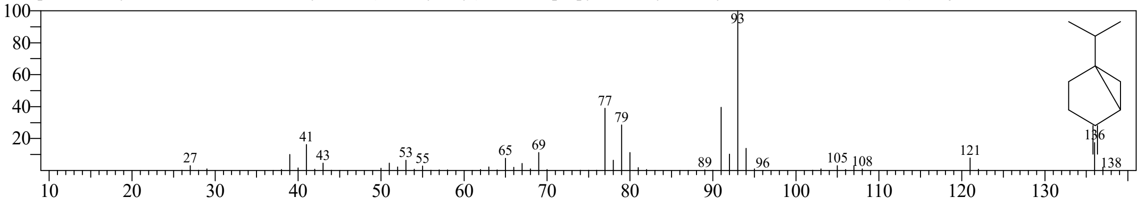

Hit#:2 Entry:11391 Library:NIST23-1.lib
SI:92 Formula:C10H16 CAS:555-10-2 MolWeight:136 RetIndex:1030
CompName:.beta.-Phellandrene $$ Cyclohexene, 3-methylene-6-(1-methylethyl)- $$ p-Mentha-1(7),2-diene $$ Phellandrene, .beta. $$ 3-Isopropyl-6-methyl

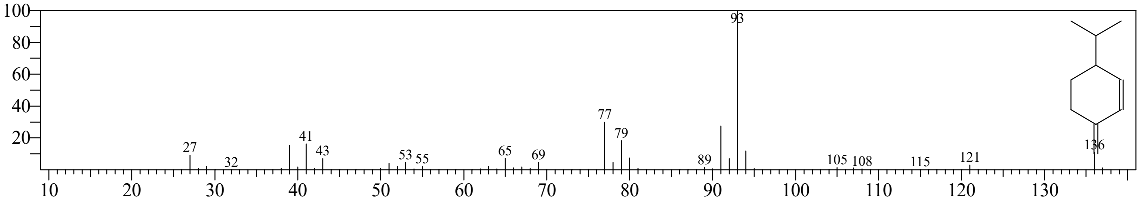

Hit#:3 Entry:8423 Library:NIST23s.lib
SI:91 Formula:C10H16 CAS:499-97-8 MolWeight:136 RetIndex:1009
CompName:Cyclohexane, 1-methylene-4-(1-methylethenyl)- $$ p-Mentha-1(7),8-diene $$ .psi.-Limonene $$ Pseudolimonen $$ Pseudolimonene $$ 1(7), 8-

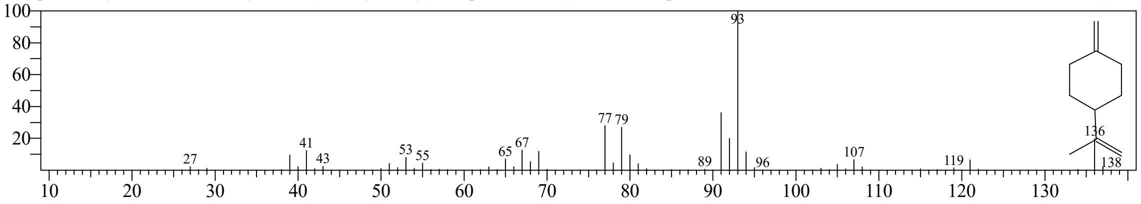

Hit#:4 Entry:11390 Library:NIST23-1.lib
SI:90 Formula:C10H16 CAS:28634-89-1 MolWeight:136 RetIndex:924
CompName:Bicyclo[3.1.0]hex-2-ene, 4-methyl-1-(1-methylethyl)- $$ 2-Thujene $$ .beta.-Thujene $$ 1-Isopropyl-4-methylbicyclo[3.1.0]hex-2-ene $$

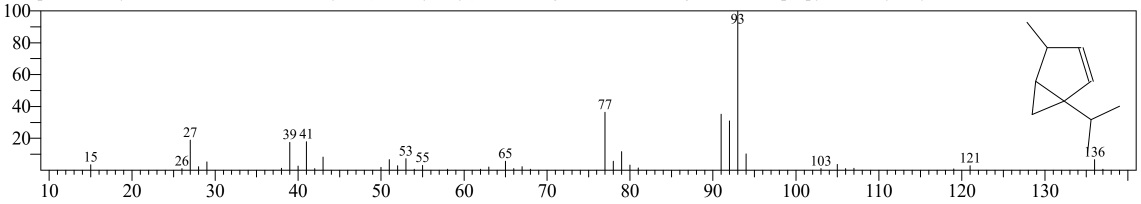

Hit#:5 Entry:11389 Library:NIST23-1.lib
SI:90 Formula:C10H16 CAS:99-84-3 MolWeight:136 RetIndex:1024
CompName:Cyclohexene, 4-methylene-1-(1-methylethyl)- $$ p-Mentha-1(7),3-diene $$ .beta.-Terpinen $$ .beta.-Terpinene $$ Beta terpinene $$ 1-Isopropy

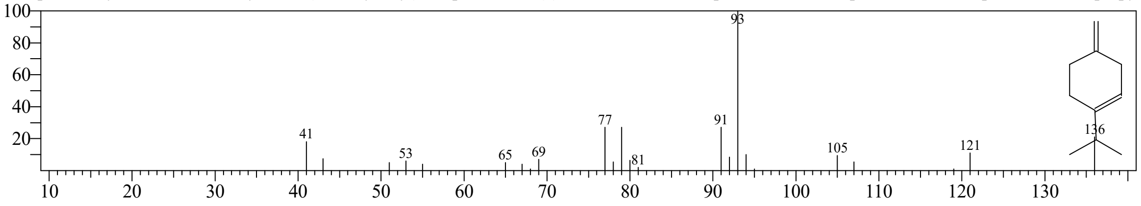

<< Target >>
Line#:4 R.Time:8.760(Scan#:1153) MassPeaks:27
RawMode:Averaged 8.755-8.765(1152-1154) BasePeak:93.10(46276)
BG Mode:Calc. from Peak Group 1 - Event 1 Scan

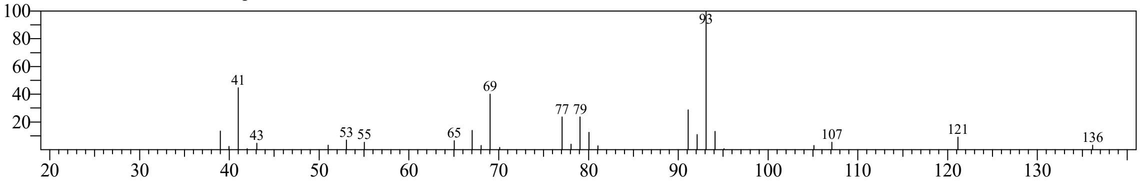

Hit#:1 Entry:8389 Library:NIST23s.lib
SI:95 Formula:C10H16 CAS:18172-67-3 MolWeight:136 RetIndex:978
CompName:Bicyclo[3.1.1]heptane, 6,6-dimethyl-2-methylene-, (1S)- $$ 2(10)-Pinene, (1S,5S)-(-)- $$ (-)-.beta.-Pinene $$ (-)-2(10)-Pinene $$ L-.beta.-Piner

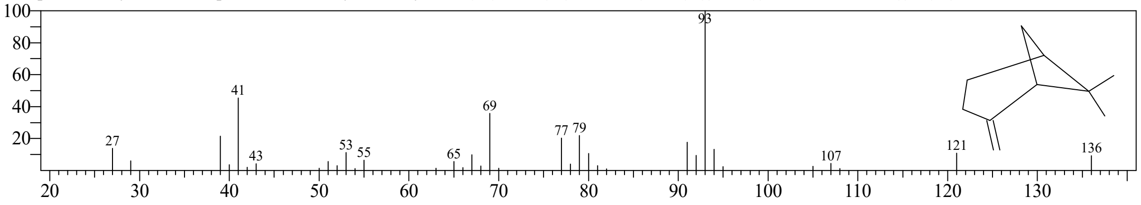

Hit#:2 Entry:8392 Library:NIST23s.lib
SI:94 Formula:C10H16 CAS:127-91-3 MolWeight:136 RetIndex:978
CompName:.beta.-Pinene $$ Bicyclo[3.1.1]heptane, 6,6-dimethyl-2-methylene- $$ 2(10)-Pinene $$ Nopinen $$ Nopinene $$ Pseudopinen $$ Pseudopinene

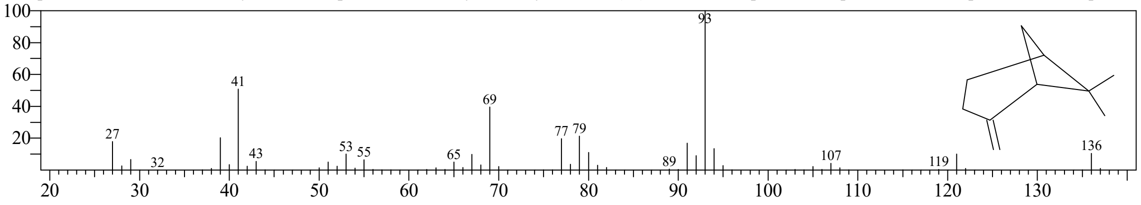

Hit#:3 Entry:8396 Library:NIST23s.lib
SI:91 Formula:C10H16 CAS:3387-41-5 MolWeight:136 RetIndex:968
CompName:Bicyclo[3.1.0]hexane, 4-methylene-1-(1-methylethyl)- $$ 1-Isopropyl-4-methylenebicyclo[3.1.0]hexane $$ 4(10)-Thujene $$ Sabinen $$ Sabin

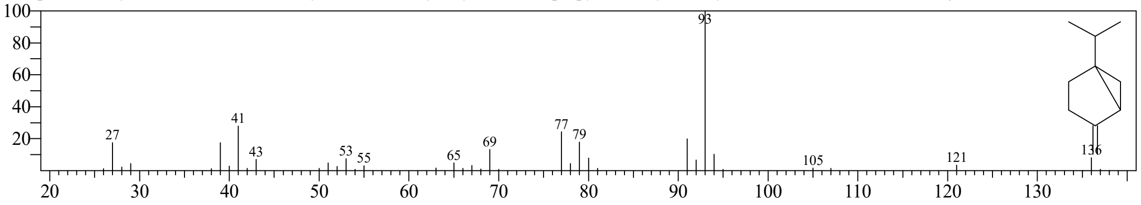

Hit#:4 Entry:11388 Library:NIST23-1.lib
SI:90 Formula:C10H16 CAS:16626-39-4 MolWeight:136 RetIndex:946
CompName:5,5-Dimethyl-1-vinylbicyclo[2.1.1]hexane $$ Bicyclo[2.1.1]hexane, 1-ethenyl-5,5-dimethyl- $$ Bicyclo[2.1.1]hexane, 5,5-dimethyl-1-vinyl- $$

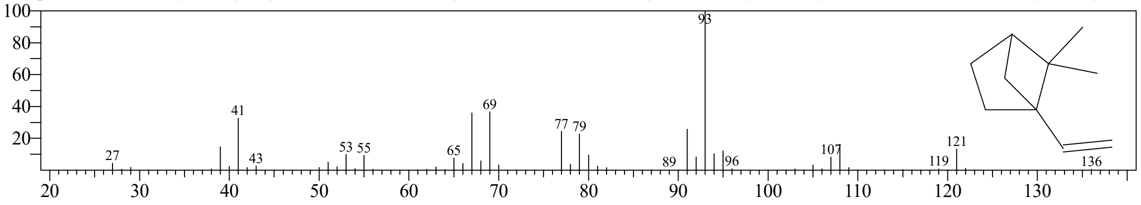

Hit#:5 Entry:8406 Library:NIST23s.lib
SI:90 Formula:C10H16 CAS:123-35-3 MolWeight:136 RetIndex:993
CompName:.beta.-Myrcene $$ 1,6-Octadiene, 7-methyl-3-methylene- $$ Myrcene $$ 7-Methyl-3-methylene-1,6-octadiene $$ 7-Methyl-3-methyleneoctadie

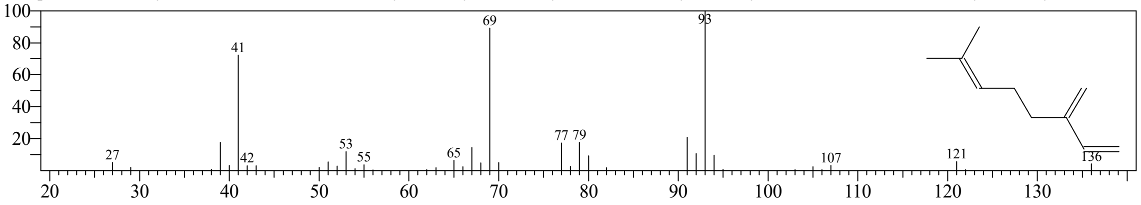

<< Target >>
Line#:5   R.Time:9.075(Scan#:1216) MassPeaks:10
RawMode:Averaged 9.070-9.080(1215-1217) BasePeak:41.00(9935)
BG Mode:Calc. from Peak Group 1 - Event 1 Scan

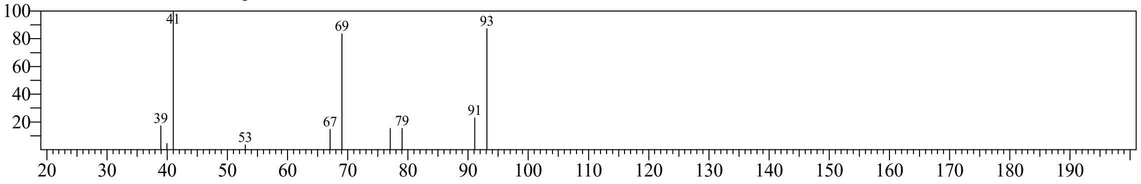

Hit#:1 Entry:8395 Library:NIST23s.lib
SI:86 Formula:C10H16 CAS:123-35-3 MolWeight:136 RetIndex:993
CompName:.beta.-Myrcene $$ 1,6-Octadiene, 7-methyl-3-methylene- $$ Myrcene $$ 7-Methyl-3-methylene-1,6-octadiene $$ 7-Methyl-3-methyleneoctadie

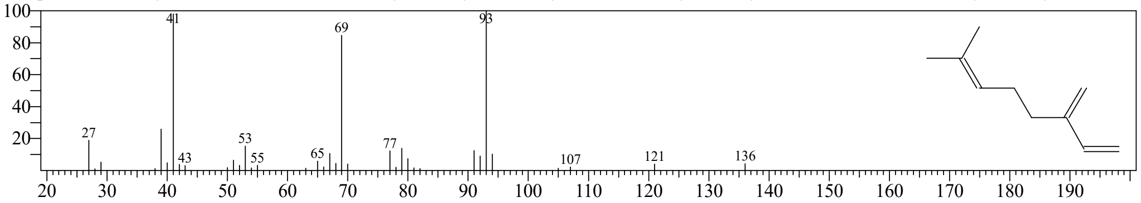

Hit#:2 Entry:58400 Library:NIST23-1.lib
SI:83 Formula:C10H16S2 CAS:73188-23-5 MolWeight:200 RetIndex:1622
CompName:4-(4-Methylpent-3-enyl)-3,6-dihydro-1,2-dithiin $$ 4-(4-Methyl-3-pentenyl)-3,6-dihydro-1,2-dithiine # $$

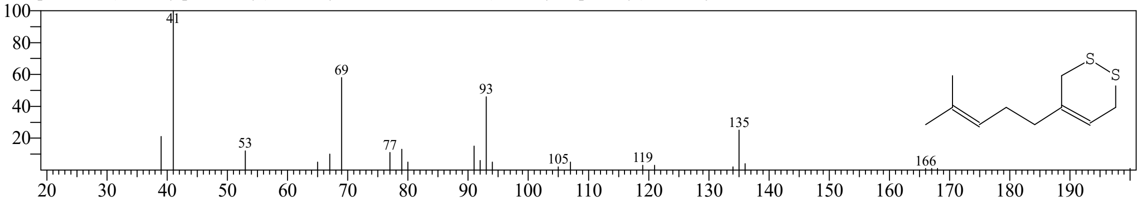

Hit#:3 Entry:25391 Library:NIST23-1.lib
SI:80 Formula:C10H11NO CAS:6580-95-6 MolWeight:161 RetIndex:1471
CompName:Ethanone, 1-cyclopropyl-2-(4-pyridinyl)- $$ Ketone, cyclopropyl 4-pyridylmethyl $$ Cyclopropyl 4-picolyl ketone $$ 1-Cyclopropyl-2-(4-pyrid

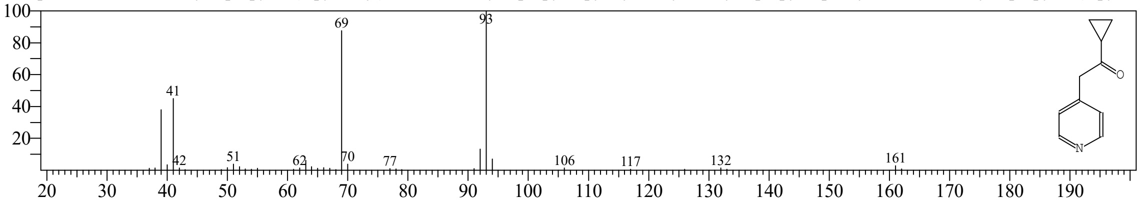

Hit#:4 Entry:3484 Library:NIST23-1.lib
SI:80 Formula:C8H14 CAS:13643-06-6 MolWeight:110 RetIndex:766
CompName:1,6-Heptadiene, 2-methyl- $$ 6-Methyl-1,6-heptadiene $$ CH2=CH(CH2)3C(CH3)=CH2 $$ 2-Methyl-1,6-heptadiene # $$

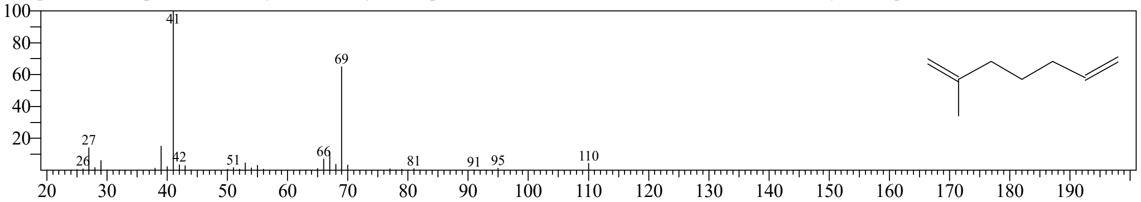

Hit#:5 Entry:25390 Library:NIST23-1.lib
SI:78 Formula:C10H11NO CAS:57276-33-2 MolWeight:161 RetIndex:1472
CompName:Ethanone, 1-cyclopropyl-2-(3-pyridinyl)- $$ Cyclopropyl 3-picolyl ketone $$ 1-Cyclopropyl-2-(3-pyridinyl)ethanone # $$

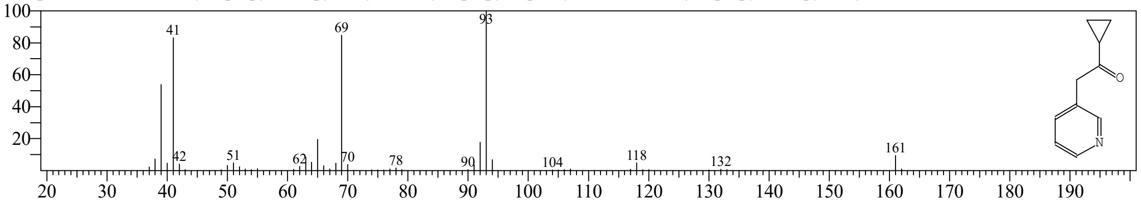

<< Target >>
Line#:6 R.Time:9.590(Scan#:1319) MassPeaks:5
RawMode:Averaged 9.585-9.595(1318-1320) BasePeak:93.10(4551)
BG Mode:Calc. from Peak Group 1 - Event 1 Scan

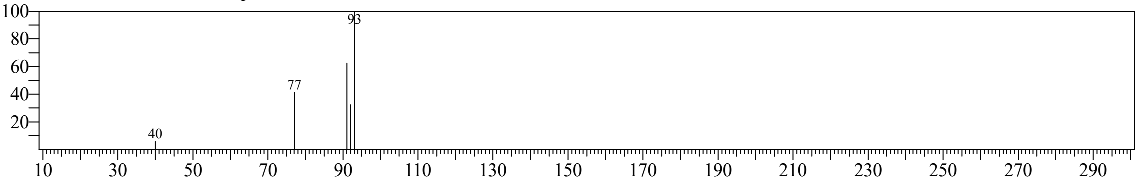

Hit#:1 Entry:172340 Library:NIST23-1.lib
SI:81 Formula:C13H12N2O4S CAS:0-00-0 MolWeight:292 RetIndex:2584
CompName:Bicyclo[2.2.1]hept-2-ene, 2-[(2,4-dinitrophenyl)thio]- $$ 2-[(2,4-dinitrophenyl)thio]bicyclo[2.2.1]hept-2-ene $$ bicyclo[2.2.1]hept-2-en-2-yl 2,

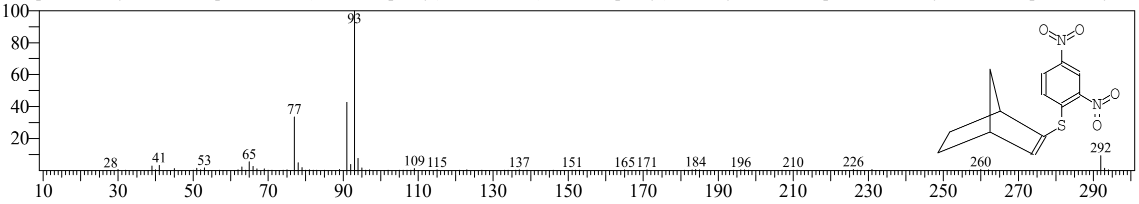

Hit#:2 Entry:8437 Library:NIST23s.lib
SI:81 Formula:C10H16 CAS:99-83-2 MolWeight:136 RetIndex:1017
CompName:.alpha.-Phellandrene $$ 1,3-Cyclohexadiene, 2-methyl-5-(1-methylethyl)- $$ .alpha.-Fellandrene $$ p-Mentha-1,5-diene $$ 5-Isopropyl-2-methy

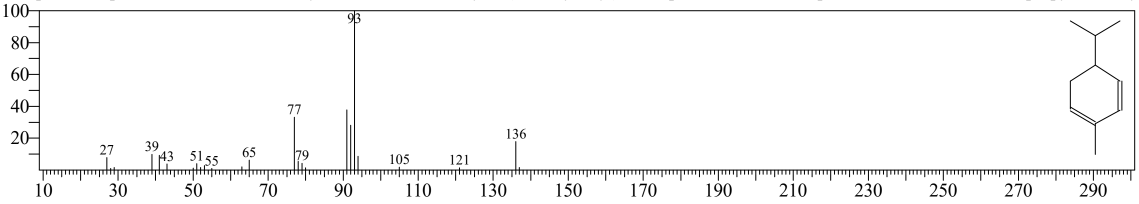

Hit#:3 Entry:11134 Library:NIST23-1.lib
SI:79 Formula:C8H12N2 CAS:109746-10-3 MolWeight:136 RetIndex:1191
CompName:1,4-Methano-1H-Cyclopropa[d]pyridazine, 4,4a,5,5a-tetrahydro-6,6-dimethyl-, (1.alpha.,4.alpha.,4a.alpha.,5a.alpha.)-

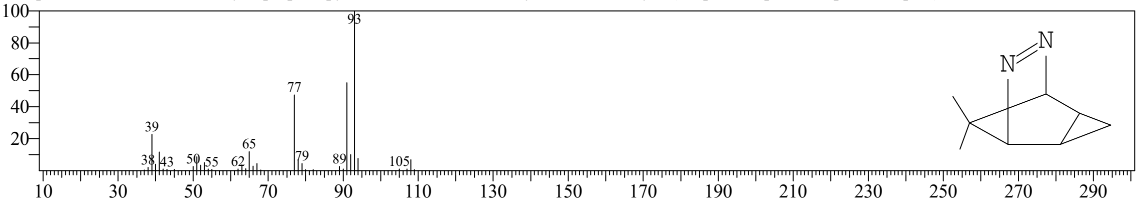

Hit#:4 Entry:8410 Library:NIST23s.lib
SI:79 Formula:C10H16 CAS:2867-05-2 MolWeight:136 RetIndex:939
CompName:Bicyclo[3.1.0]hex-2-ene, 2-methyl-5-(1-methylethyl)- $$ 3-Thujene $$ .alpha.-Thujene $$ Thujene, .alpha.- $$ Origanene $$ 5-Isopropyl-2-met

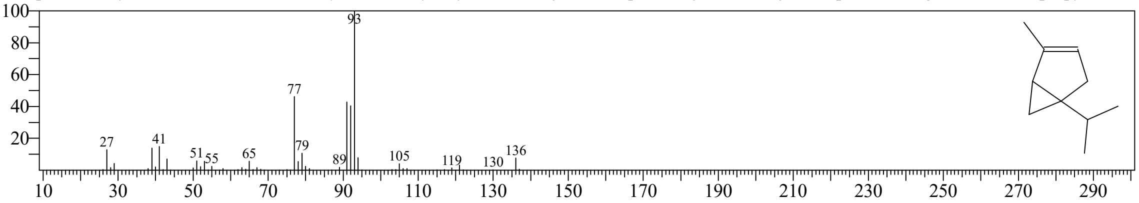

Hit#:5 Entry:11390 Library:NIST23-1.lib
SI:76 Formula:C10H16 CAS:28634-89-1 MolWeight:136 RetIndex:924
CompName:Bicyclo[3.1.0]hex-2-ene, 4-methyl-1-(1-methylethyl)- $$ 2-Thujene $$ .beta.-Thujene $$ 1-Isopropyl-4-methylbicyclo[3.1.0]hex-2-ene $$

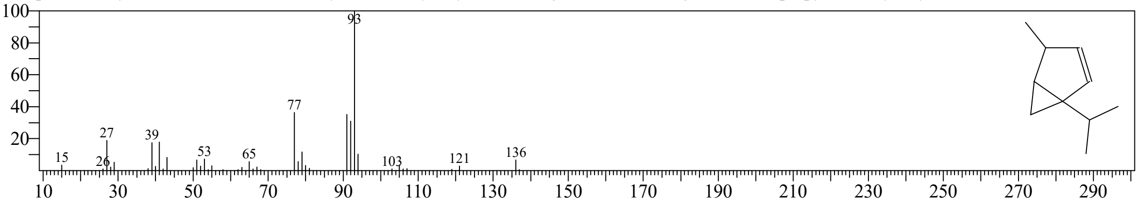

<< Target >>
Line#:7 R.Time:9.680(Scan#:1337) MassPeaks:7
RawMode:Averaged 9.675-9.685(1336-1338) BasePeak:93.10(5011)
BG Mode:Calc. from Peak Group 1 - Event 1 Scan

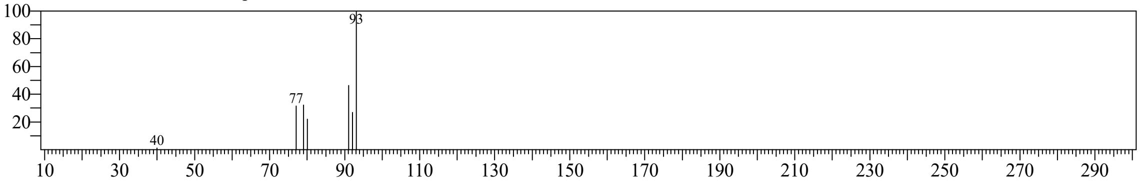

Hit#:1 Entry:8437 Library:NIST23s.lib
SI:81 Formula:C10H16 CAS:99-83-2 MolWeight:136 RetIndex:1017
CompName:.alpha.-Phellandrene $$ 1,3-Cyclohexadiene, 2-methyl-5-(1-methylethyl)- $$ .alpha.-Fellandrene $$ p-Mentha-1,5-diene $$ 5-Isopropyl-2-methy

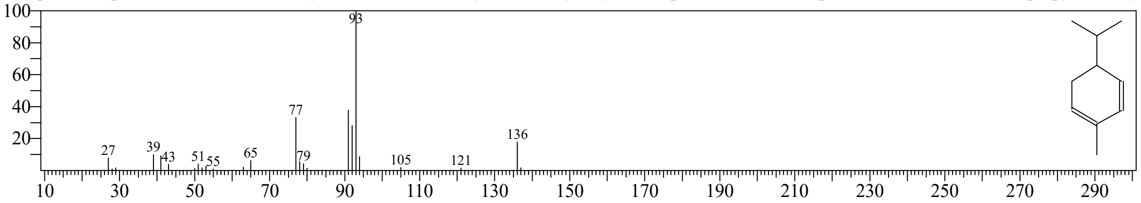

Hit#:2 Entry:8430 Library:NIST23s.lib
SI:79 Formula:C10H16 CAS:2867-05-2 MolWeight:136 RetIndex:939
CompName:Bicyclo[3.1.0]hex-2-ene, 2-methyl-5-(1-methylethyl)- $$ 3-Thujene $$ .alpha.-Thujene $$ Thujene, .alpha.- $$ Origanene $$ 5-Isopropyl-2-met

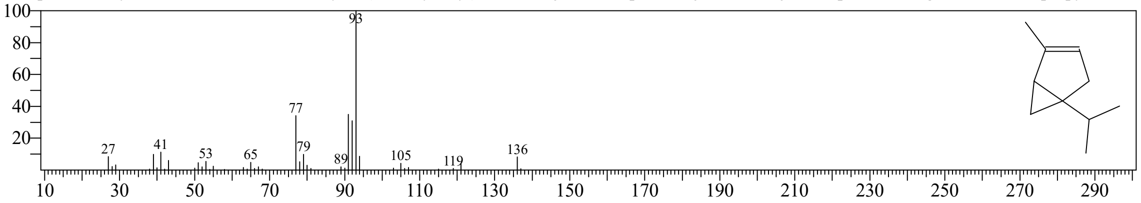

Hit#:3 Entry:172340 Library:NIST23-1.lib
SI:78 Formula:C13H12N2O4S CAS:0-00-0 MolWeight:292 RetIndex:2584
CompName:Bicyclo[2.2.1]hept-2-ene, 2-[(2,4-dinitrophenyl)thio]- $$ 2-[(2,4-dinitrophenyl)thio]bicyclo[2.2.1]hept-2-ene $$ bicyclo[2.2.1]hept-2-en-2-yl 2,

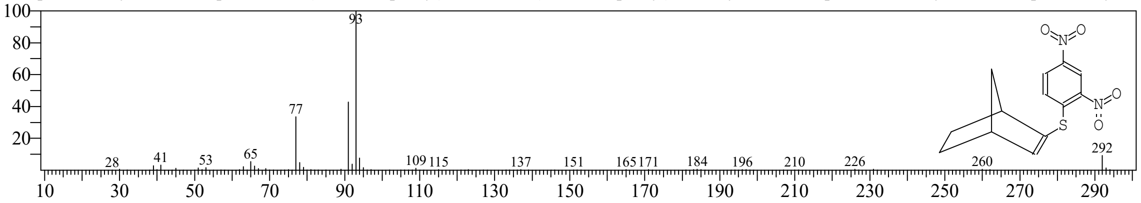

Hit#:4 Entry:11390 Library:NIST23-1.lib
SI:78 Formula:C10H16 CAS:28634-89-1 MolWeight:136 RetIndex:924
CompName:Bicyclo[3.1.0]hex-2-ene, 4-methyl-1-(1-methylethyl)- $$ 2-Thujene $$ .beta.-Thujene $$ 1-Isopropyl-4-methylbicyclo[3.1.0]hex-2-ene $$

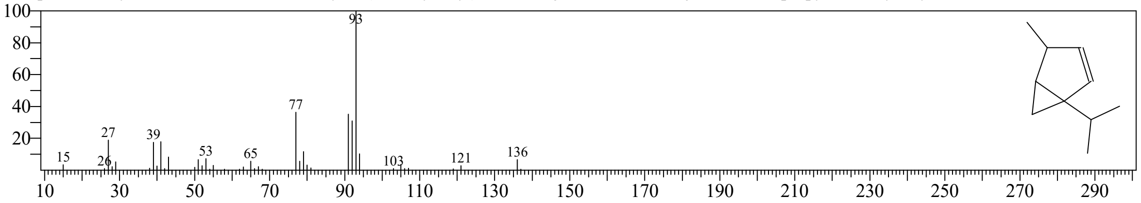

Hit#:5 Entry:3193 Library:NIST23s.lib
SI:77 Formula:C8H12 CAS:54211-14-2 MolWeight:108 RetIndex:846
CompName:Bicyclo[4.1.0]heptane, 7-methylene- $$ 7-Methylenenorcarane $$ 7-Methylenebicyclo[4.1.0]heptane $$

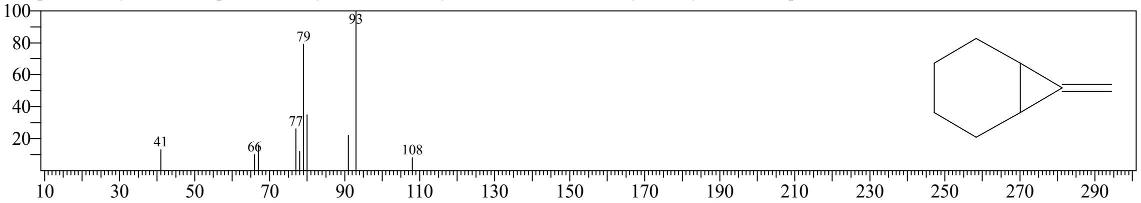

<< Target >>
Line#:8   R.Time:9.915(Scan#:1384) MassPeaks:9
RawMode:Averaged 9.910-9.920(1383-1385) BasePeak:93.10(5574)
BG Mode:Calc. from Peak Group 1 - Event 1 Scan

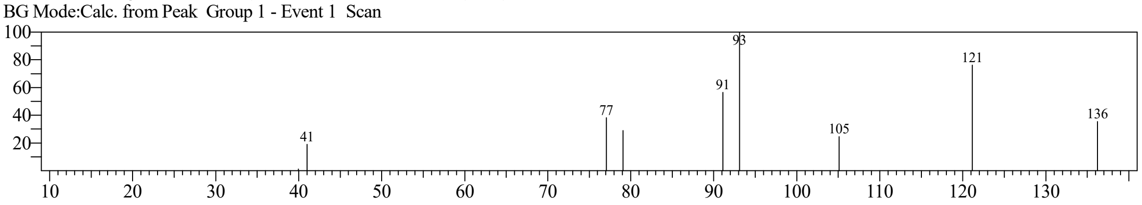

Hit#:1 Entry:8478 Library:NIST23s.lib
SI:79 Formula:C10H16 CAS:586-63-0 MolWeight:136 RetIndex:1083
CompName:Cyclohexene, 3-methyl-6-(1-methylethylidene)- $$ p-Mentha-2,4(8)-diene $$ Isoterpinolene $$ 3-Methyl-6-(1-methylethylidene)-1-cyclohexene

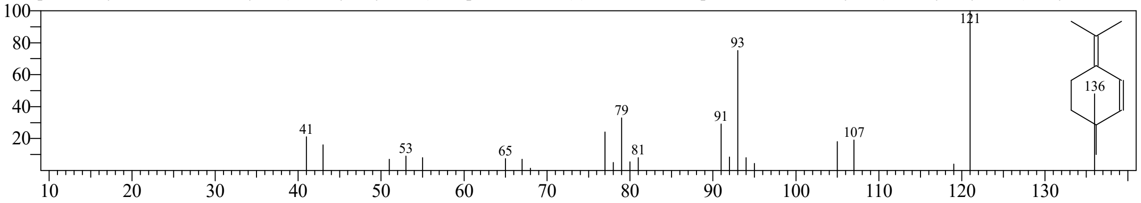

Hit#:2 Entry:8472 Library:NIST23s.lib
SI:79 Formula:C10H16 CAS:99-86-5 MolWeight:136 RetIndex:1036
CompName:1,3-Cyclohexadiene, 1-methyl-4-(1-methylethyl)- $$ .alpha.-Terpinene $$ .alpha.-Terpinen $$ p-Mentha-1,3-diene $$ Terpilene $$ 1-Isopropyl-4

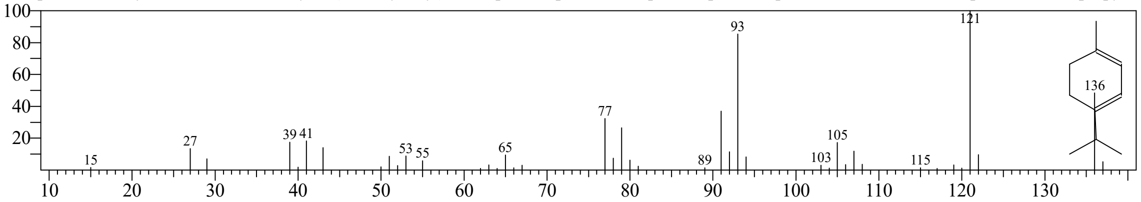

Hit#:3 Entry:11428 Library:NIST23-1.lib
SI:79 Formula:C10H16 CAS:29050-33-7 MolWeight:136 RetIndex:970
CompName:(+)-4-Carene $$ 4,7,7-Trimethylbicyclo[4.1.0]hept-2-ene # $$

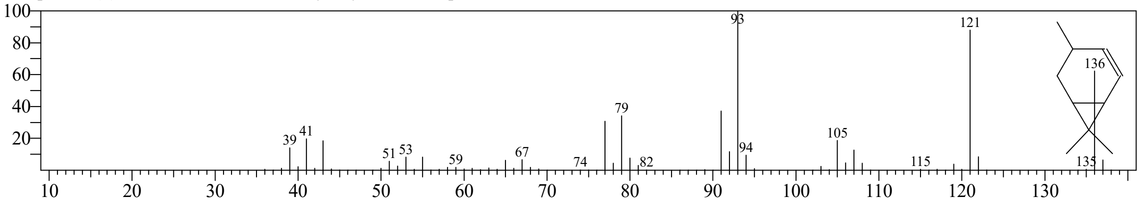

Hit#:4 Entry:11417 Library:NIST23-1.lib
SI:79 Formula:C10H16 CAS:4497-92-1 MolWeight:136 RetIndex:985
CompName:Bicyclo[4.1.0]hept-2-ene, 3,7,7-trimethyl-, (1S-cis)-

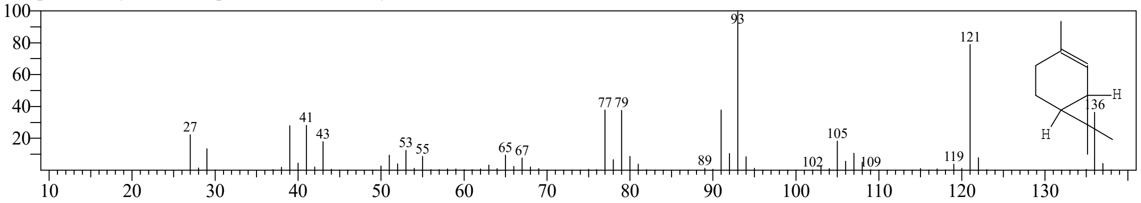

Hit#:5 Entry:11389 Library:NIST23-1.lib
SI:79 Formula:C10H16 CAS:99-84-3 MolWeight:136 RetIndex:1024
CompName:Cyclohexene, 4-methylene-1-(1-methylethyl)- $$ p-Mentha-1(7),3-diene $$ .beta.-Terpinen $$ .beta.-Terpinene $$ Beta terpinene $$ 1-Isopropy

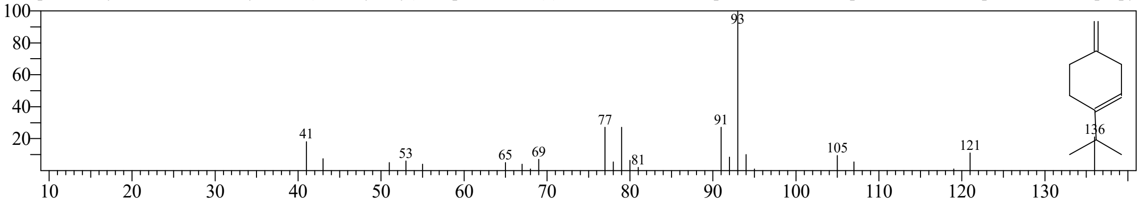

<< Target >>
Line#:9   R.Time:10.140(Scan#:1429) MassPeaks:10
RawMode:Averaged 10.135-10.145(1428-1430) BasePeak:119.10(13502)
BG Mode:Calc. from Peak Group 1 - Event 1 Scan

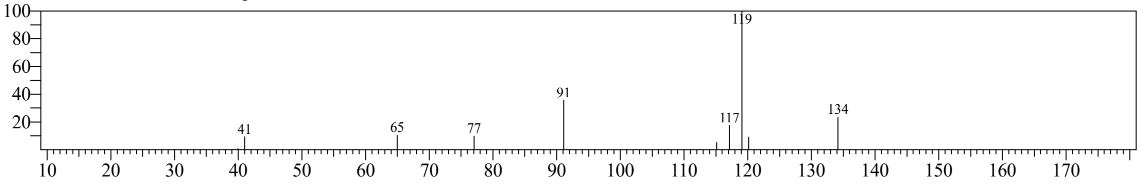

Hit#:1 Entry:7837 Library:NIST23s.lib
SI:87 Formula:C10H14 CAS:99-87-6 MolWeight:134 RetIndex:1017
CompName:p-Cymene $$ Benzene, 1-methyl-4-(1-methylethyl)- $$ p-Cimene $$ p-Cymol $$ p-Isopropyltoluene $$ p-Methylisopropylbenzene $$ Campho

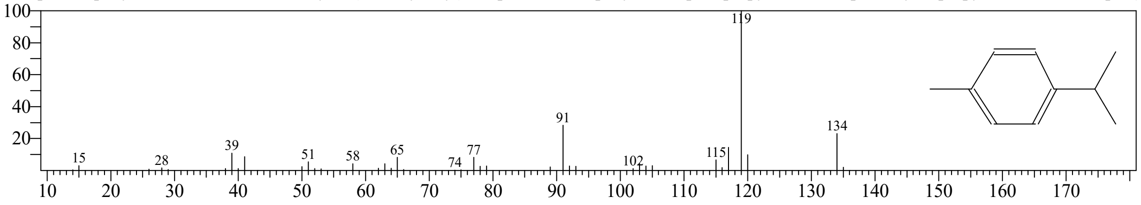

Hit#:2 Entry:7841 Library:NIST23s.lib
SI:85 Formula:C10H14 CAS:18368-95-1 MolWeight:134 RetIndex:1098
CompName:1,3,8-p-Menthatriene $$ p-Mentha-1,3,8-triene $$ 1-Isopropenyl-4-methyl-1,3-cyclohexadiene # $$ 1,3,8-para-Menthatriene $$ p-1,3,8-Mentha

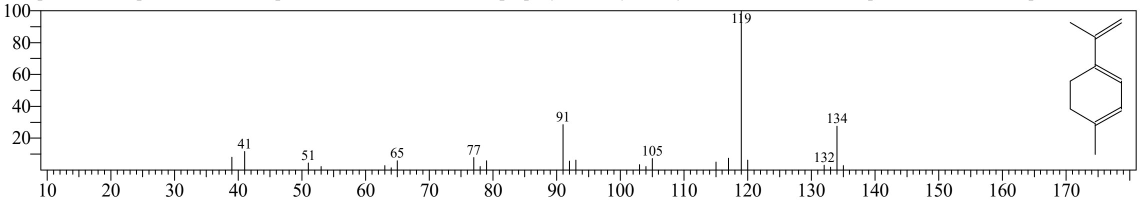

Hit#:3 Entry:7874 Library:NIST23s.lib
SI:85 Formula:C10H14 CAS:527-84-4 MolWeight:134 RetIndex:1022
CompName:o-Cymene $$ Benzene, 1-methyl-2-(1-methylethyl)- $$ o-Cymol $$ o-Isopropyltoluene $$ 1-Isopropyl-2-methylbenzene $$ 1-Methyl-2-isoprop

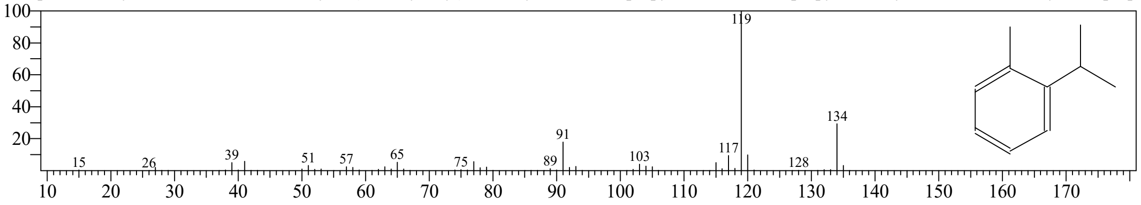

Hit#:4 Entry:18332 Library:NIST23s.lib
SI:84 Formula:C12H16O CAS:1671-77-8 MolWeight:176 RetIndex:1480
CompName:1-Pentanone, 1-(4-methylphenyl)- $$ 4-Methyl-1-pentanoylbenzene $$ 1-(4-Methylphenyl)-1-pentanone # $$

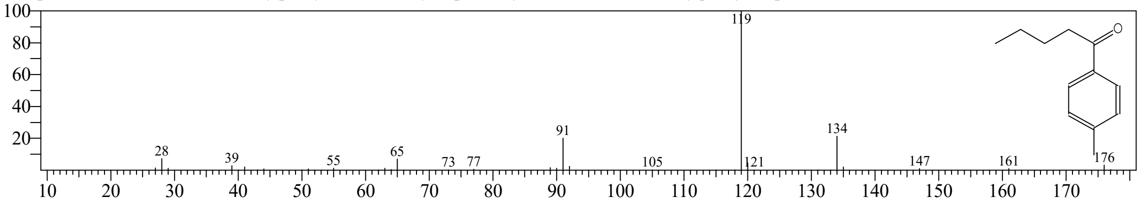

Hit#:5 Entry:10603 Library:NIST23-1.lib
SI:84 Formula:C10H14 CAS:535-77-3 MolWeight:134 RetIndex:1013
CompName:Benzene, 1-methyl-3-(1-methylethyl)- $$ m-Cymene $$ .beta.-Cymene $$ m-Cymol $$ m-Isopropyltoluene $$ m-Methylisopropylbenzene $$ 1-

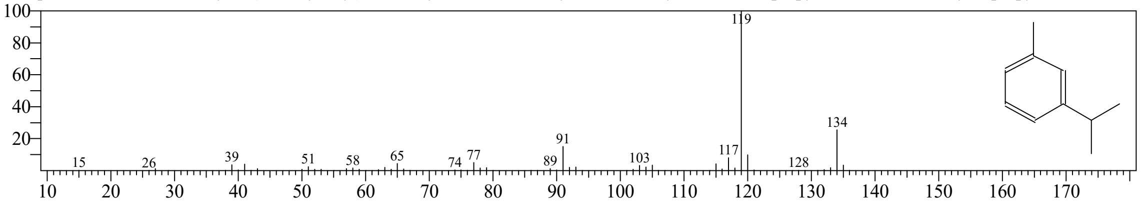

<< Target >>
Line#:10 R.Time:10.290(Scan#:1459) MassPeaks:22
RawMode:Averaged 10.285-10.295(1458-1460) BasePeak:68.05(20592)
BG Mode:Calc. from Peak Group 1 - Event 1 Scan

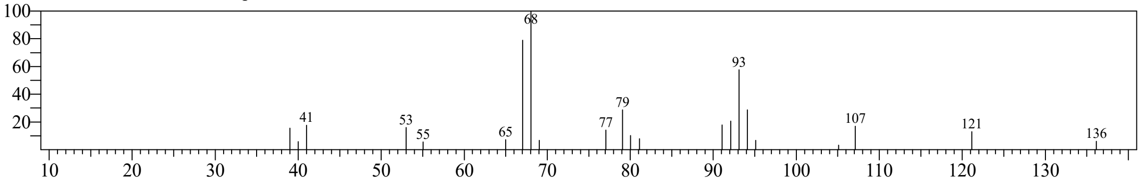

Hit#:1 Entry:11346 Library:NIST23-1.lib
SI:91 Formula:C10H16 CAS:5989-27-5 MolWeight:136 RetIndex:1031
CompName:D-Limonene $$ Cyclohexene, 1-methyl-4-(1-methylethenyl)-, (R)- $$ p-Mentha-1,8-diene, (R)-(+)- $$ (+)-(R)-Limonene $$ (+)-(4R)-Limonene

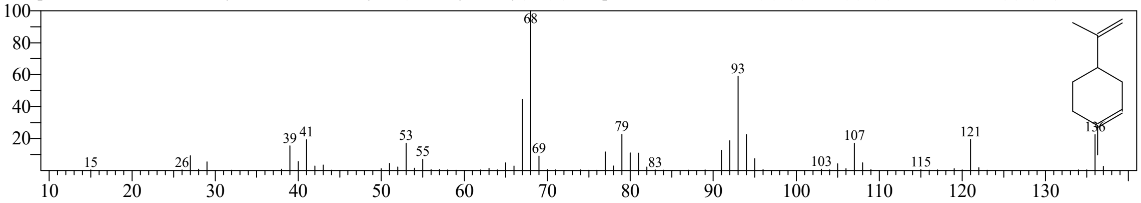

Hit#:2 Entry:8363 Library:NIST23s.lib
SI:90 Formula:C10H16 CAS:138-86-3 MolWeight:136 RetIndex:1031
CompName:Limonene $$ Cyclohexene, 1-methyl-4-(1-methylethenyl)- $$ p-Mentha-1,8-diene $$ .alpha.-Limonene $$ Cajeputen $$ Cajeputene $$ Cinen $

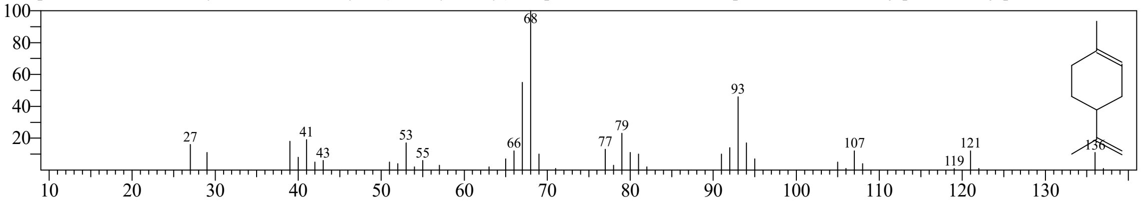

Hit#:3 Entry:8365 Library:NIST23s.lib
SI:89 Formula:C10H16 CAS:5989-54-8 MolWeight:136 RetIndex:1031
CompName:Cyclohexene, 1-methyl-4-(1-methylethenyl)-, (S)- $$ p-Mentha-1,8-diene, (S)-(-)- $$ (-)-Limonene $$ L-Limonene $$ Limonene $$ 4-Isoproper

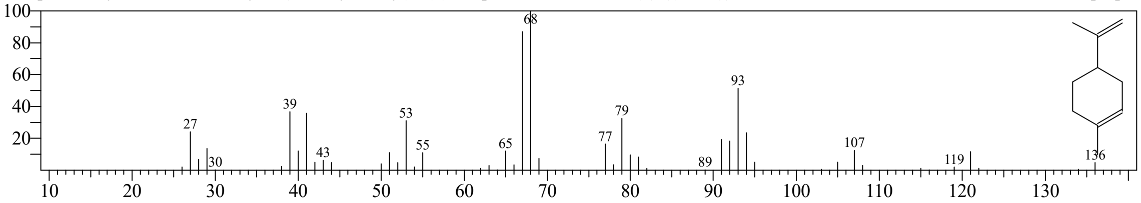

Hit#:4 Entry:11343 Library:NIST23-1.lib
SI:89 Formula:C10H16 CAS:0-00-0 MolWeight:136 RetIndex:981
CompName:Cyclobutane, 1,3-diisopropenyl-, trans $$ 1,3-Diisopropenylcyclobutane # $$

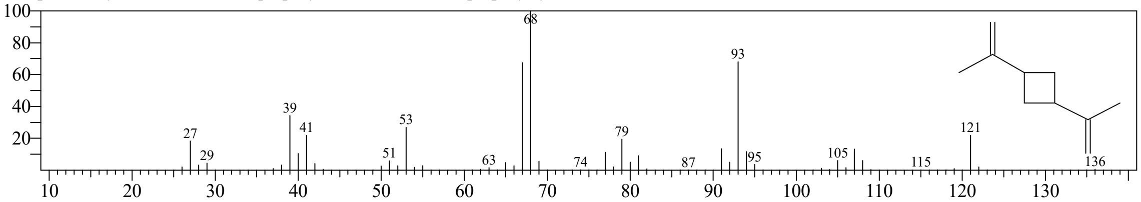

Hit#:5 Entry:11344 Library:NIST23-1.lib
SI:88 Formula:C10H16 CAS:1743-61-9 MolWeight:136 RetIndex:990
CompName:Cyclohexene, 4-ethenyl-1,4-dimethyl- $$ 1,4-Dimethyl-4-vinylcyclohexene $$ 1,4-Dimethyl-4-vinyl-1-cyclohexene # $$ 1,4-Dimethyl-4-etheny

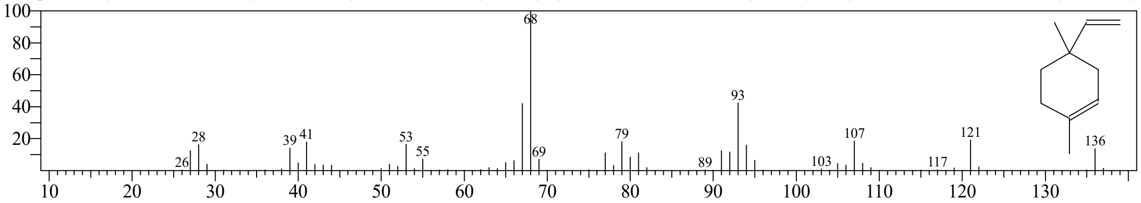

<< Target >>
Line#:11 R.Time:10.405(Scan#:1482) MassPeaks:8
RawMode:Averaged 10.400-10.410(1481-1483) BasePeak:43.00(2759)
BG Mode:Calc. from Peak Group 1 - Event 1 Scan

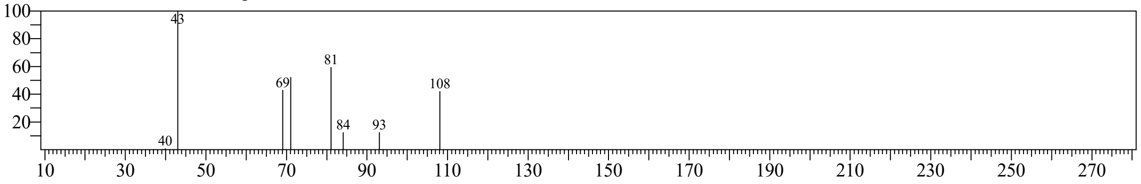

Hit#:1 Entry:126803 Library:NIST23-1.lib
SI:71 Formula:C10H11BrOS CAS:0-00-0 MolWeight:258 RetIndex:1638
CompName:3-Bromothiophenol, S-(2-methylpropionyl)-

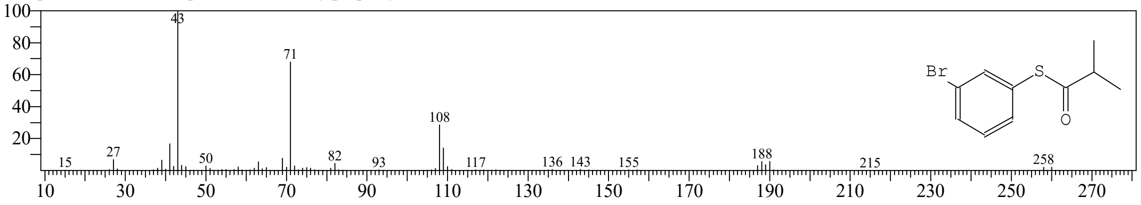

Hit#:2 Entry:126802 Library:NIST23-1.lib
SI:70 Formula:C10H11BrOS CAS:0-00-0 MolWeight:258 RetIndex:1619
CompName:2-Bromothiophenol, S-(2-methylpropionyl)-

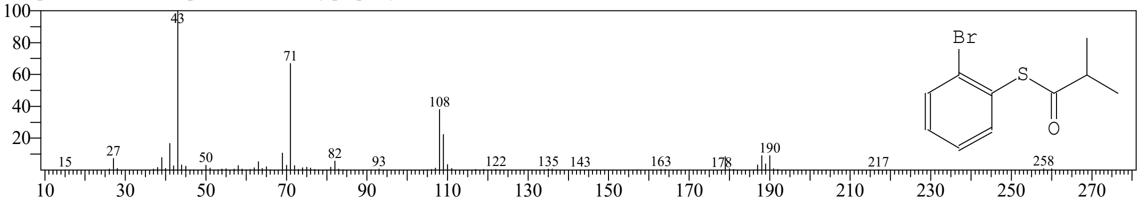

Hit#:3 Entry:86324 Library:NIST23-1.lib
SI:69 Formula:C10H11NO3S CAS:0-00-0 MolWeight:225 RetIndex:1777
CompName:4-Nitrothiophenol, S-(2-methylpropionyl)-

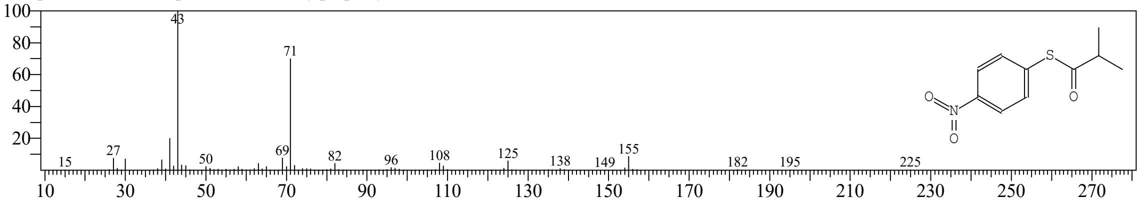

Hit#:4 Entry:33627 Library:NIST23-1.lib
SI:69 Formula:C9H16O3 CAS:58371-97-4 MolWeight:172 RetIndex:1255
CompName:Butanoic acid, 4-oxopentyl ester $$ 4-Oxopentyl butyrate # $$

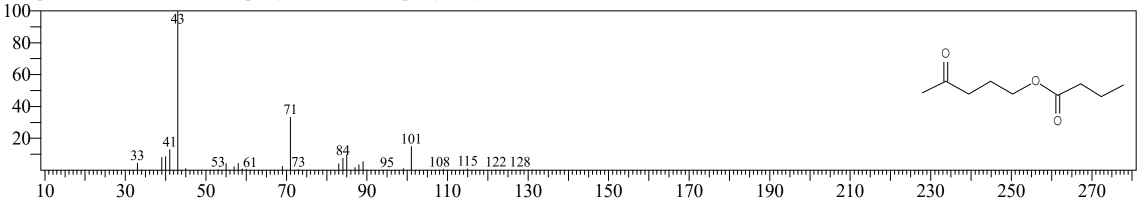

Hit#:5 Entry:153729 Library:NIST23-1.lib
SI:69 Formula:C15H18O5 CAS:176379-72-9 MolWeight:278 RetIndex:1914
CompName:3,4-Dihydroxybenzaldehyde, bis(2-methylpropionate)

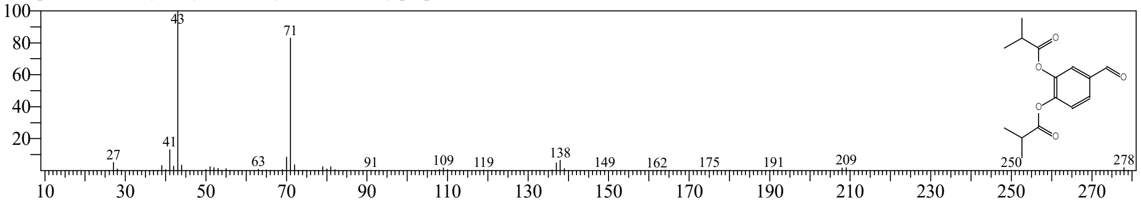

<< Target >>
Line#:12 R.Time:11.170(Scan#:1635) MassPeaks:17
RawMode:Averaged 11.165-11.175(1634-1636) BasePeak:93.10(12933)
BG Mode:Calc. from Peak Group 1 - Event 1 Scan

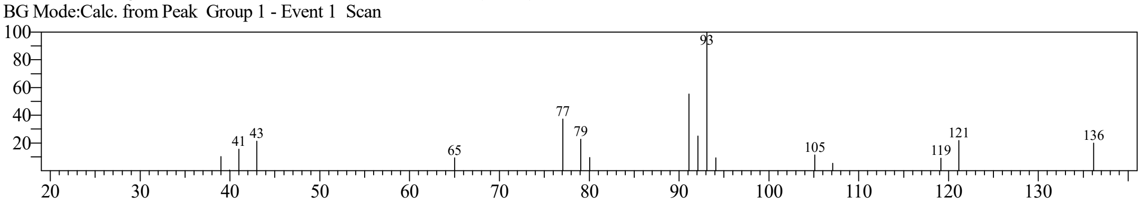

Hit#:1 Entry:8426 Library:NIST23s.lib
SI:89 Formula:C10H16 CAS:99-85-4 MolWeight:136 RetIndex:1038
CompName:.gamma.-Terpinene $$ 1,4-Cyclohexadiene, 1-methyl-4-(1-methylethyl)- $$ .gamma.-Terpinen $$ p-Mentha-1,4-diene $$ Crithmene $$ Moslen

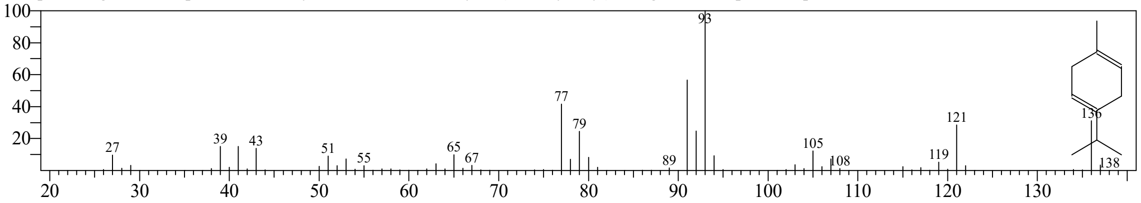

Hit#:2 Entry:11406 Library:NIST23-1.lib
SI:87 Formula:C10H16 CAS:2867-05-2 MolWeight:136 RetIndex:939
CompName:Bicyclo[3.1.0]hex-2-ene, 2-methyl-5-(1-methylethyl)- $$ 3-Thujene $$ .alpha.-Thujene $$ Thujene, .alpha.- $$ Origanene $$ 5-Isopropyl-2-met

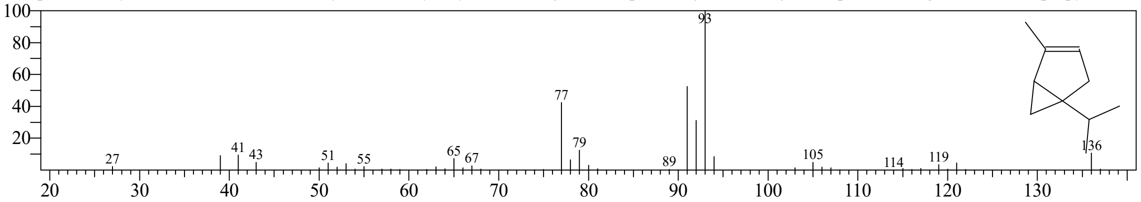

Hit#:3 Entry:8437 Library:NIST23s.lib
SI:87 Formula:C10H16 CAS:99-83-2 MolWeight:136 RetIndex:1017
CompName:.alpha.-Phellandrene $$ 1,3-Cyclohexadiene, 2-methyl-5-(1-methylethyl)- $$ .alpha.-Fellandrene $$ p-Mentha-1,5-diene $$ 5-Isopropyl-2-methy

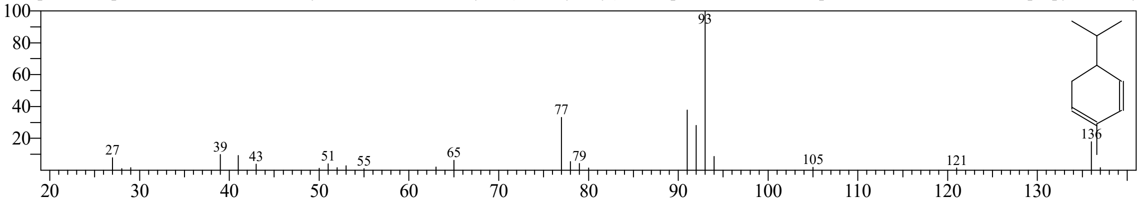

Hit#:4 Entry:8441 Library:NIST23s.lib
SI:87 Formula:C10H16 CAS:13466-78-9 MolWeight:136 RetIndex:992
CompName:3-Carene $$ Bicyclo[4.1.0]hept-3-ene, 3,7,7-trimethyl- $$ delta-3-Carene $$ 3,7,7-Trimethylbicyclo[4.1.0]hept-3-ene $$ .delta. 3-carene $$ 3,7

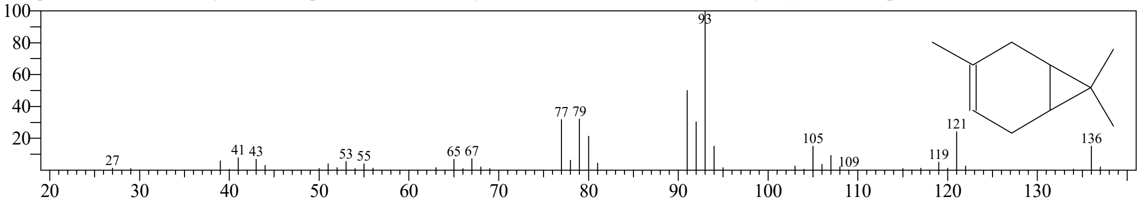

Hit#:5 Entry:11389 Library:NIST23-1.lib
SI:86 Formula:C10H16 CAS:99-84-3 MolWeight:136 RetIndex:1024
CompName:Cyclohexene, 4-methylene-1-(1-methylethyl)- $$ p-Mentha-1(7),3-diene $$ .beta.-Terpinen $$ .beta.-Terpinene $$ Beta terpinene $$ 1-Isopropy

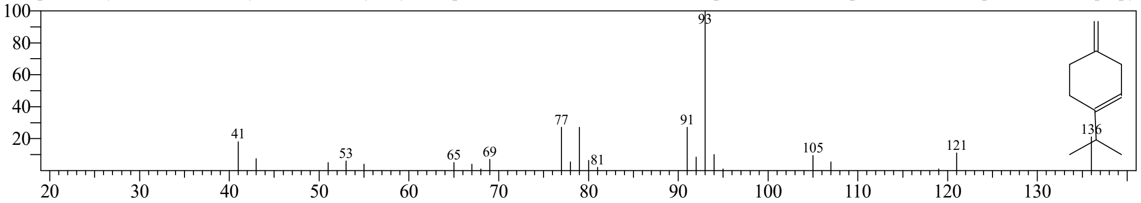

<< Target >>
Line#:13   R.Time:12.005(Scan#:1802) MassPeaks:10
RawMode:Averaged 12.000-12.010(1801-1803) BasePeak:93.10(5507)
BG Mode:Calc. from Peak Group 1 - Event 1 Scan

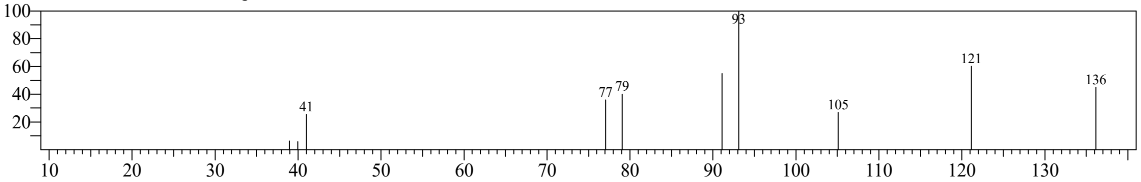

Hit#:1 Entry:11428 Library:NIST23-1.lib
SI:79 Formula:C10H16 CAS:29050-33-7 MolWeight:136 RetIndex:970
CompName:(+)-4-Carene $$ 4,7,7-Trimethylbicyclo[4.1.0]hept-2-ene # $$

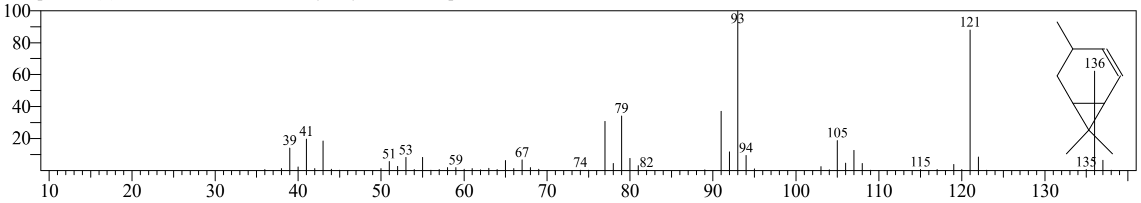

Hit#:2 Entry:8446 Library:NIST23s.lib
SI:78 Formula:C10H16 CAS:99-85-4 MolWeight:136 RetIndex:1038
CompName:.gamma.-Terpinene $$ 1,4-Cyclohexadiene, 1-methyl-4-(1-methylethyl)- $$ .gamma.-Terpinen $$ p-Mentha-1,4-diene $$ Crithmene $$ Moslene

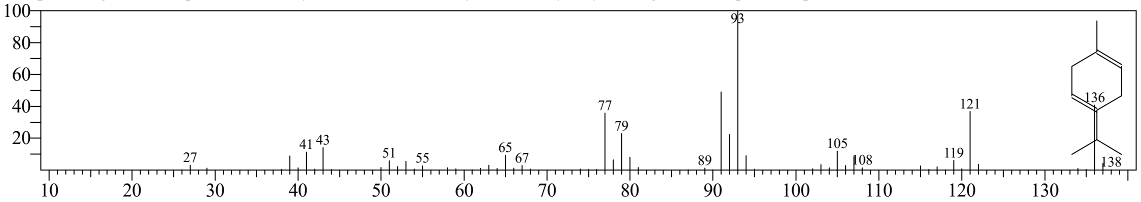

Hit#:3 Entry:11417 Library:NIST23-1.lib
SI:78 Formula:C10H16 CAS:4497-92-1 MolWeight:136 RetIndex:985
CompName:Bicyclo[4.1.0]hept-2-ene, 3,7,7-trimethyl-, (1S-cis)-

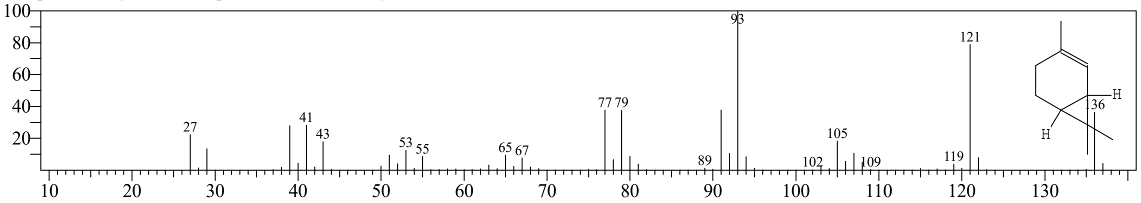

Hit#:4 Entry:8478 Library:NIST23s.lib
SI:78 Formula:C10H16 CAS:586-63-0 MolWeight:136 RetIndex:1083
CompName:Cyclohexene, 3-methyl-6-(1-methylethylidene)- $$ p-Mentha-2,4(8)-diene $$ Isoterpinolene $$ 3-Methyl-6-(1-methylethylidene)-1-cyclohexene

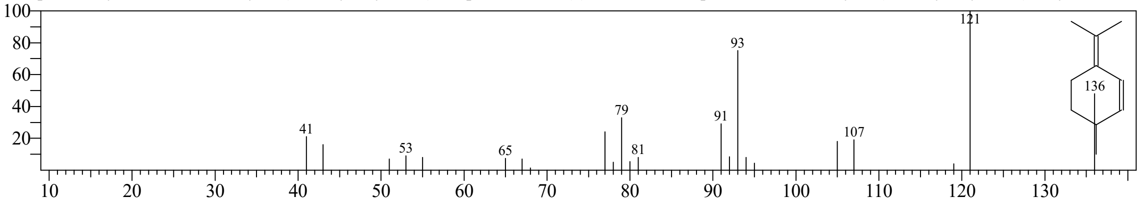

Hit#:5 Entry:8472 Library:NIST23s.lib
SI:78 Formula:C10H16 CAS:99-86-5 MolWeight:136 RetIndex:1036
CompName:1,3-Cyclohexadiene, 1-methyl-4-(1-methylethyl)- $$ .alpha.-Terpinene $$ .alpha.-Terpinen $$ p-Mentha-1,3-diene $$ Terpilene $$ 1-Isopropyl-4

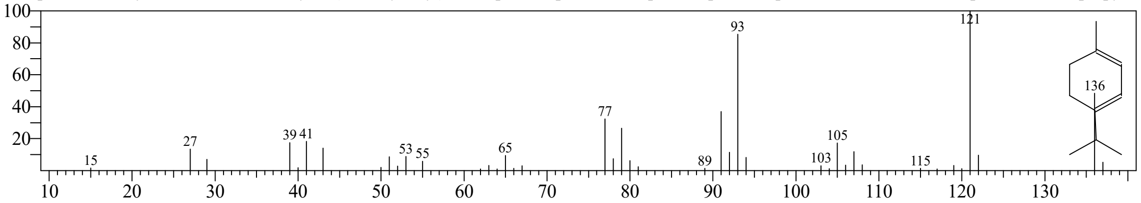

<< Target >>
Line#:14 R.Time:14.860(Scan#:2373) MassPeaks:28
RawMode:Averaged 14.855-14.865(2372-2374) BasePeak:71.05(24072)
BG Mode:Calc. from Peak Group 1 - Event 1 Scan

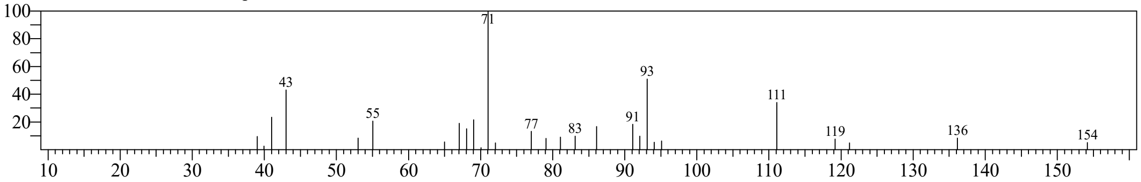

Hit#:1 Entry:21057 Library:NIST23-1.lib
SI:90 Formula:C10H18O CAS:562-74-3 MolWeight:154 RetIndex:1184
CompName:Terpinen-4-ol $$ 3-Cyclohexen-1-ol, 4-methyl-1-(1-methylethyl)- $$ p-Menth-1-en-4-ol $$ 1-Terpinen-4-ol $$ 4-Carvomenthenol $$ 4-Terpine

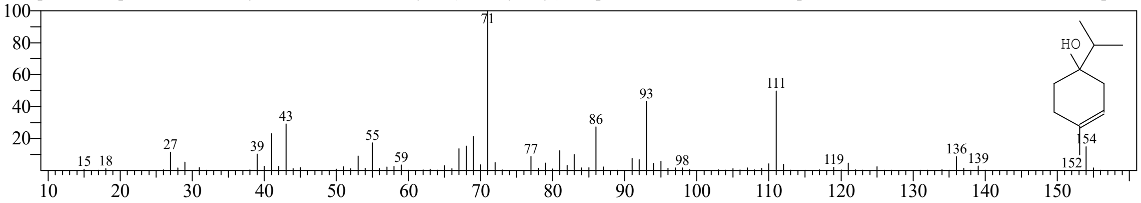

Hit#:2 Entry:21056 Library:NIST23-1.lib
SI:90 Formula:C10H18O CAS:20126-76-5 MolWeight:154 RetIndex:1184
CompName:3-Cyclohexen-1-ol, 4-methyl-1-(1-methylethyl)-, (R)- $$ p-Menth-1-en-4-ol, (R)-(-)- $$ (-)-Terpinen-4-ol $$ (-)-4-Terpineol $$ L-terpinen-4-ol

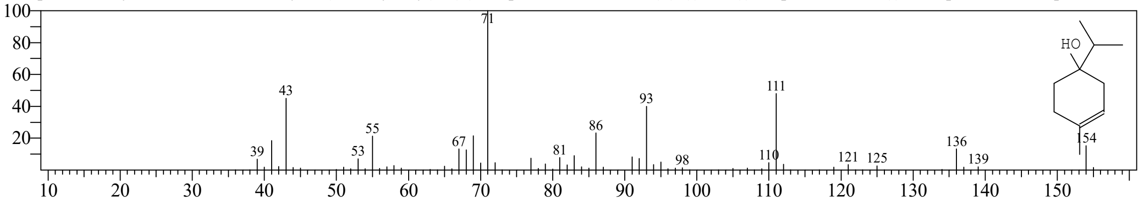

Hit#:3 Entry:12964 Library:NIST23s.lib
SI:80 Formula:C10H18O CAS:78-70-6 MolWeight:154 RetIndex:1106
CompName:Linalool $$ 1,6-Octadien-3-ol, 3,7-dimethyl- $$ .beta.-Linalool $$ Linalol $$ Linalyl alcohol $$ 2,6-Dimethyl-2,7-octadien-6-ol $$ allo-Ocimen

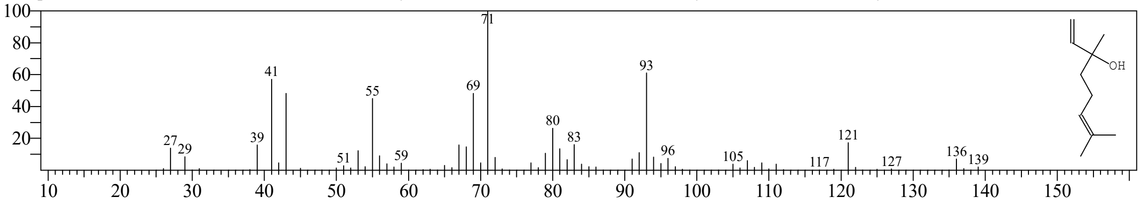

Hit#:4 Entry:21052 Library:NIST23-1.lib
SI:78 Formula:C10H18O CAS:546-79-2 MolWeight:154 RetIndex:1093
CompName:5-Isopropyl-2-methylbicyclo[3.1.0]hexan-2-ol # $$ Bicyclo[3.1.0]hexan-2-ol, 2-methyl-5-(1-methylethyl)- $$ 4-Thujanol $$ Sabinene hydrate

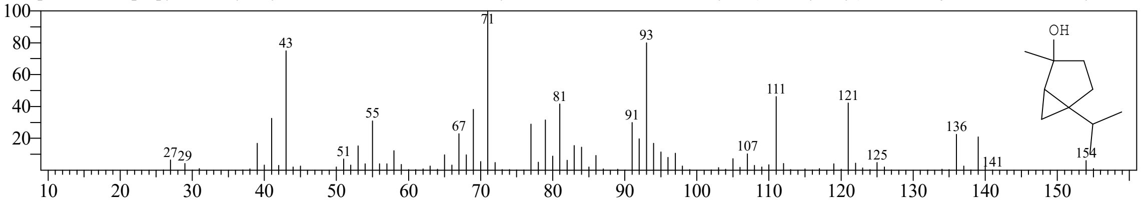

Hit#:5 Entry:12884 Library:NIST23s.lib
SI:78 Formula:C10H18O CAS:17699-16-0 MolWeight:154 RetIndex:1093
CompName:Bicyclo[3.1.0]hexan-2-ol, 2-methyl-5-(1-methylethyl)-, (1.alpha.,2.alpha.,5.alpha.)- $$ (1R,2R,5S)-5-Isopropyl-2-methylbicyclo[3.1.0]hexan-2-

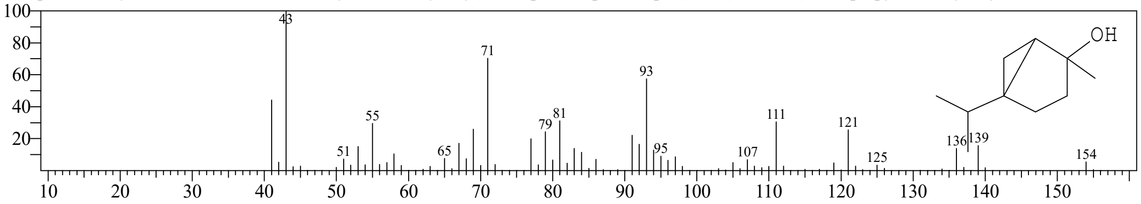

<< Target >>
Line#:15   R.Time:15.270(Scan#:2455) MassPeaks:3
RawMode:Averaged 15.265-15.275(2454-2456) BasePeak:59.05(1753)
BG Mode:Calc. from Peak Group 1 - Event 1 Scan

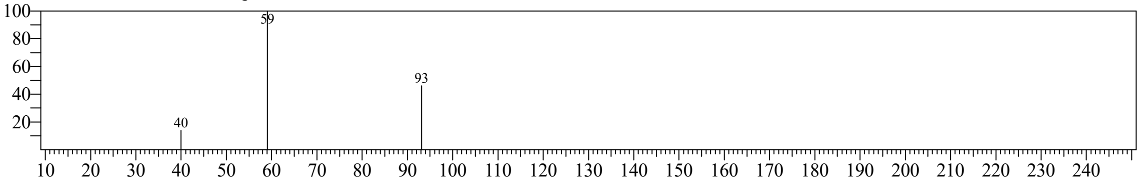

Hit#:1 Entry:41094 Library:NIST23-1.lib
SI:77 Formula:C5H11BrO2 CAS:133532-45-3 MolWeight:182 RetIndex:1097
CompName:3-Ethoxy-2-bromo-1-propanol $$ 2-Bromo-3-ethoxy-1-propanol # $$

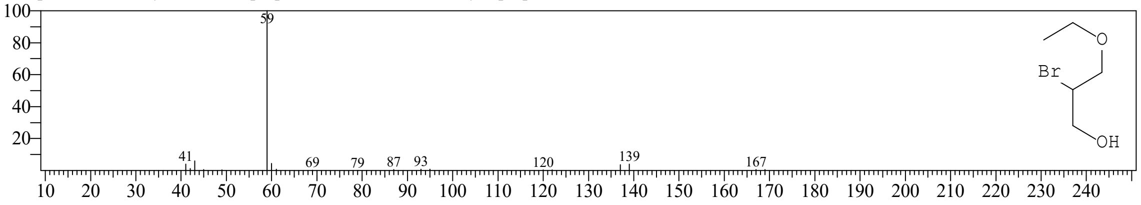

Hit#:2 Entry:155 Library:NIST23s.lib
SI:77 Formula:C2H5NO CAS:123-39-7 MolWeight:59 RetIndex:801
CompName:Formamide, N-methyl- $$ Methylformamide $$ Monomethylformamide $$ N-Methylformamide $$ HCONHCH3 $$ EK 7011 $$ NSC 3051 $

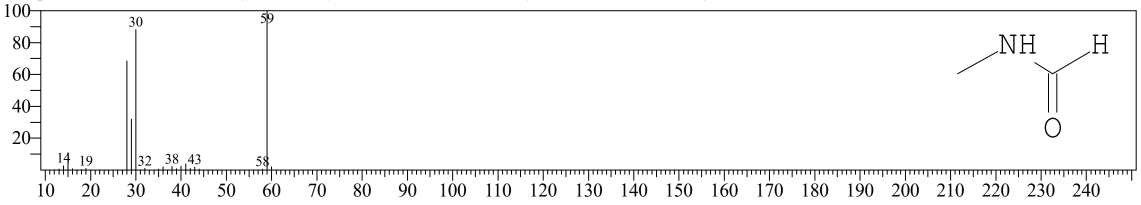

Hit#:3 Entry:17022 Library:NIST23-1.lib
SI:74 Formula:C6H12O4 CAS:0-00-0 MolWeight:148 RetIndex:1074
CompName:Hydroxymethyl 2-hydroxy-2-methylpropionate $$ 2-Hydroxyethyl 2-hydroxy-2-methylpropanoate # $$

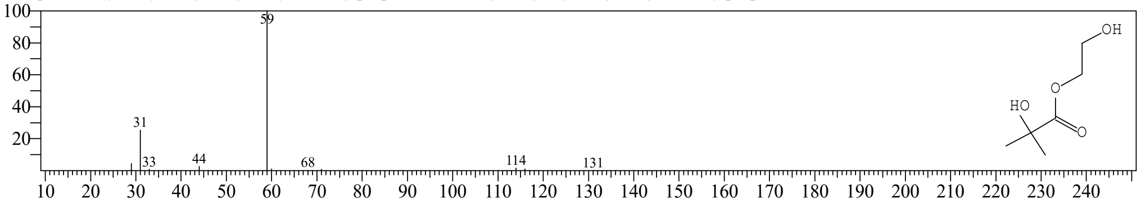

Hit#:4 Entry:202009 Library:NIST23-1.lib
SI:73 Formula:C14H18O8 CAS:0-00-0 MolWeight:314 RetIndex:2126
CompName:1,2-Benzenediol, O,O'-di(2-methoxyethoxycarbonyl)-

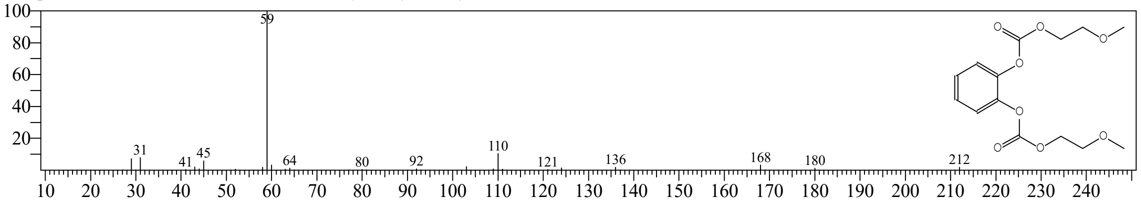

Hit#:5 Entry:113828 Library:NIST23-1.lib
SI:73 Formula:C7H9BrN2O3 CAS:296879-03-3 MolWeight:248 RetIndex:1795
CompName:Pyrimidine-2,4(1H,3H)-dione, 5-bromo-1-(1-methoxyethyl)- $$ 5-Bromo-1-(1-methoxyethyl)-2,4(1H,3H)-pyrimidinedione # $$

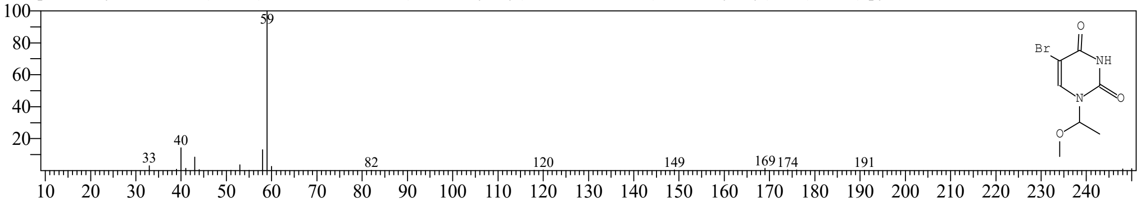

<< Target >>
Line#:16 R.Time:17.965(Scan#:2994) MassPeaks:5
RawMode:Averaged 17.960-17.970(2993-2995) BasePeak:162.10(1687)
BG Mode:Calc. from Peak Group 1 - Event 1 Scan

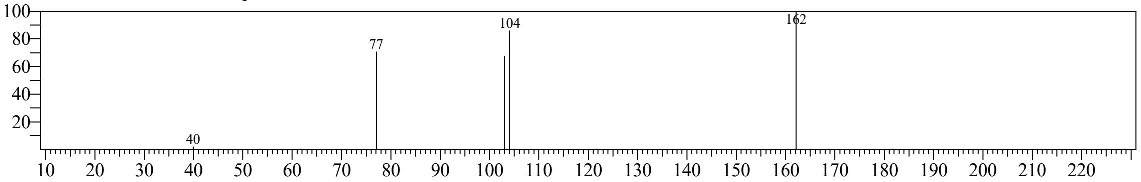

Hit#:1 Entry:25763 Library:NIST23-1.lib
SI:81 Formula:C8H6N2O2 CAS:21084-84-4 MolWeight:162 RetIndex:1752
CompName:1,2,4-Oxadiazol-3(2H)-one, 5-phenyl- $$ 1,2,4-Oxadiazol-3-ol, 5-phenyl- $$ 5-Phenyl-1,2,4-oxadiazol-3(2H)-one # $$

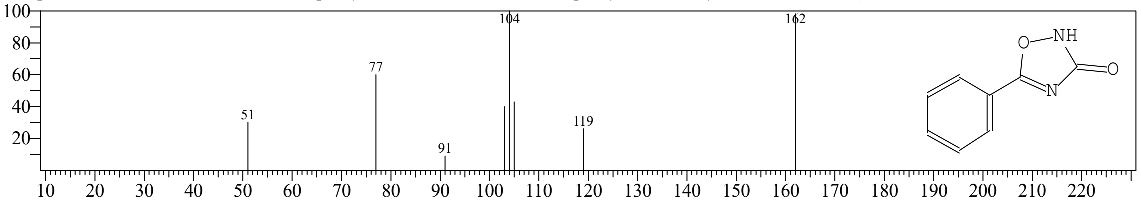

Hit#:2 Entry:25780 Library:NIST23-1.lib
SI:71 Formula:C8H6N2O2 CAS:1445-69-8 MolWeight:162 RetIndex:1849
CompName:1,4-Dioxo-1,2,3,4-tetrahydrophthalazine $$ Phthalhydrazide $$ 1,4-Phthalazinedione, 2,3-dihydro- $$ Phthalazine-1,4-dione $$ Phthalazine-1,4

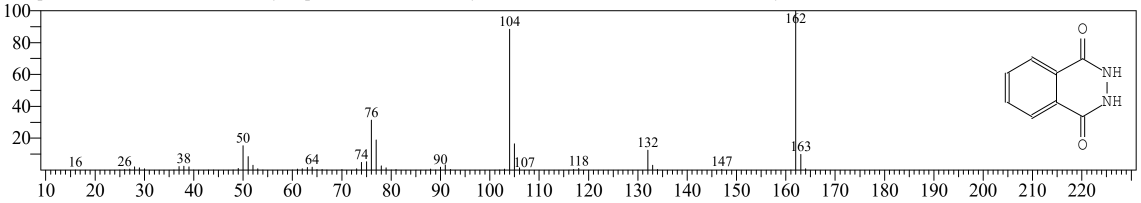

Hit#:3 Entry:25779 Library:NIST23-1.lib
SI:70 Formula:C8H6N2O2 CAS:1875-48-5 MolWeight:162 RetIndex:1634
CompName:1H-Isoindole-1,3(2H)-dione, 2-amino- $$ Phthalimide, N-amino- $$ Hydrazine, 1,1-(1,2-phenylenedicarbonyl)- $$ Hydrazine, 1,1-phthaloyl- $$

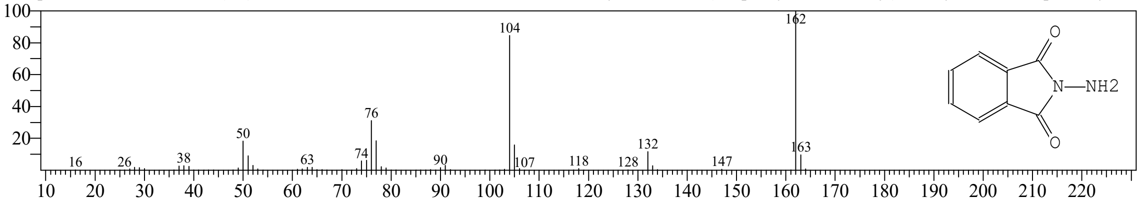

Hit#:4 Entry:79845 Library:NIST23-1.lib
SI:70 Formula:C10H8N2S2 CAS:1014-99-9 MolWeight:220 RetIndex:2120
CompName:p-Xylylene dithiocyanate

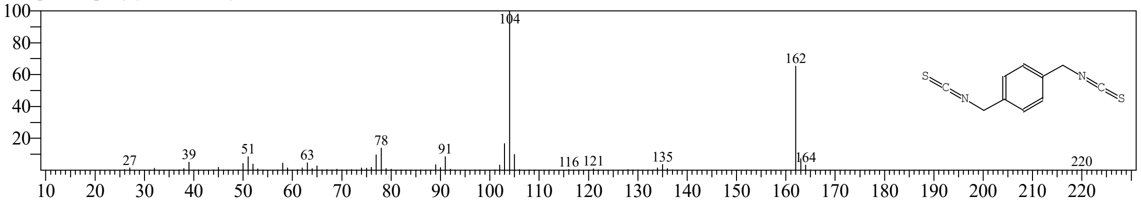

Hit#:5 Entry:25762 Library:NIST23-1.lib
SI:69 Formula:C8H6N2O2 CAS:120-06-9 MolWeight:162 RetIndex:1748
CompName:Sydnone, 3-phenyl- $$ N-Phenylsydnone $$ 3-Phenylsydnone $$ Phenylsydnone $$ 3-Phenylsydnon $$

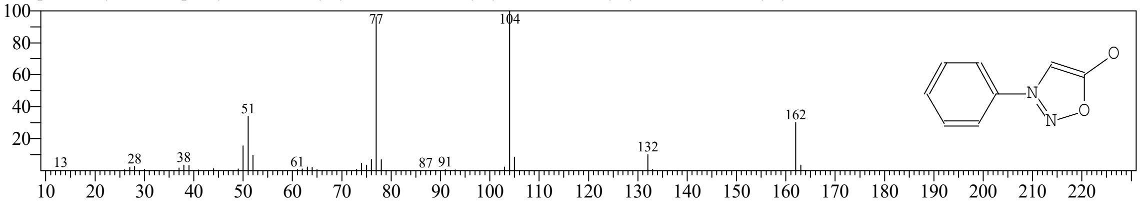

<< Target >>
Line#:17 R.Time:20.895(Scan#:3580) MassPeaks:6
RawMode:Averaged 20.890-20.900(3579-3581) BasePeak:178.10(2364)
BG Mode:Calc. from Peak Group 1 - Event 1 Scan

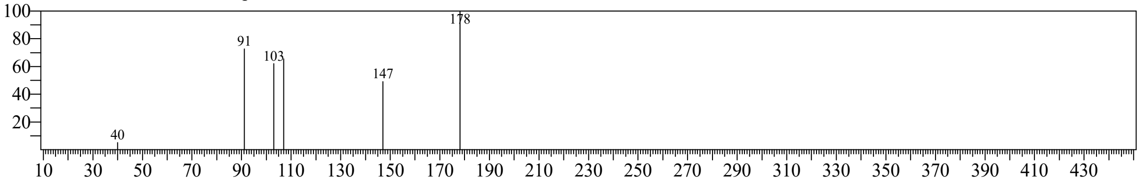

Hit#:1 Entry:142072 Library:NIST23-1.lib
SI:66 Formula:C17H19NO2 CAS:0-00-0 MolWeight:269 RetIndex:2102
CompName:Benzylphenethylamine, N-methoxycarbonyl-

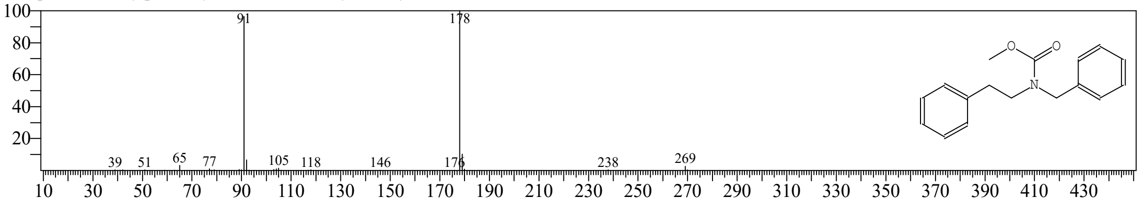

Hit#:2 Entry:6072 Library:NIST23-2.lib
SI:66 Formula:C20H20O6 CAS:0-00-0 MolWeight:356 RetIndex:2993
CompName:Dimethyl 2,4-bis(4-hydroxyphenyl)cyclobutane-1,3-dicarboxylate (isomer 2) $$ 1,3-Cyclobutanedicarboxylic acid, 2,4-bis(4-hydroxyphenyl)-, 1

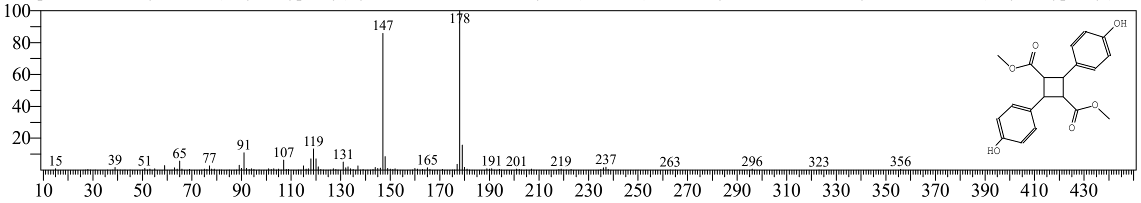

Hit#:3 Entry:67215 Library:NIST23-2.lib
SI:66 Formula:C24H24O8 CAS:0-00-0 MolWeight:440 RetIndex:3062
CompName:Dimethyl 2,4-bis(4-hydroxyphenyl)cyclobutane-1,3-dicarboxylate, diacetate (isomer 2) $$ 1,3-Cyclobutanedicarboxylic acid, 2,4-bis[4-(acetylox

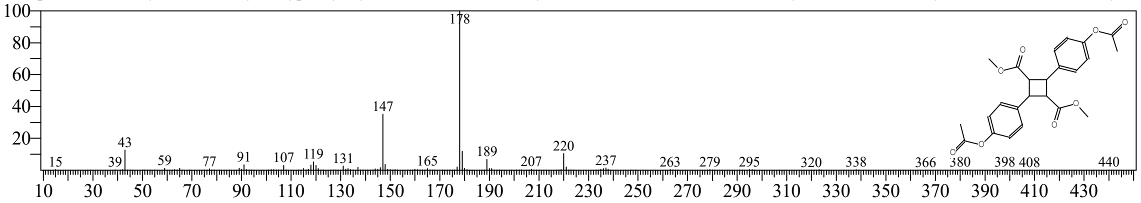

Hit#:4 Entry:185452 Library:NIST23-1.lib
SI:65 Formula:C17H19NO4 CAS:485-33-6 MolWeight:301 RetIndex:3154
CompName:dl-Laudanosoline hydrobromide $$ 6,7-Isoquinolinediol, 1-[(3,4-dihydroxyphenyl)methyl]-1,2,3,4-tetrahydro-2-methyl- $$ 1-(3,4-Dihydroxybe

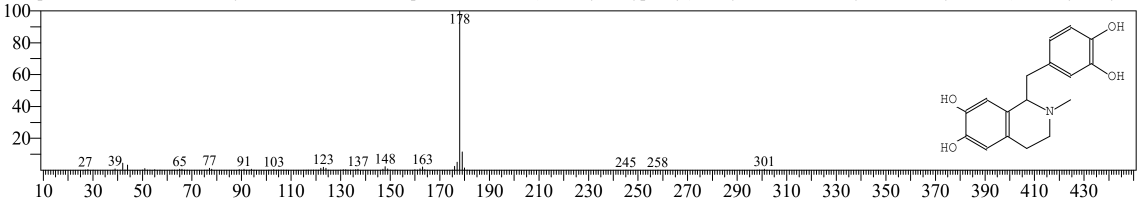

Hit#:5 Entry:38460 Library:NIST23-1.lib
SI:64 Formula:C11H14O2 CAS:93-15-2 MolWeight:178 RetIndex:1405
CompName:Methyleugenol $$ Benzene, 1,2-dimethoxy-4-(2-propenyl)- $$ Benzene, 4-allyl-1,2-dimethoxy- $$ Ent 21040 $$ Eugenol methyl ether $$ Euge

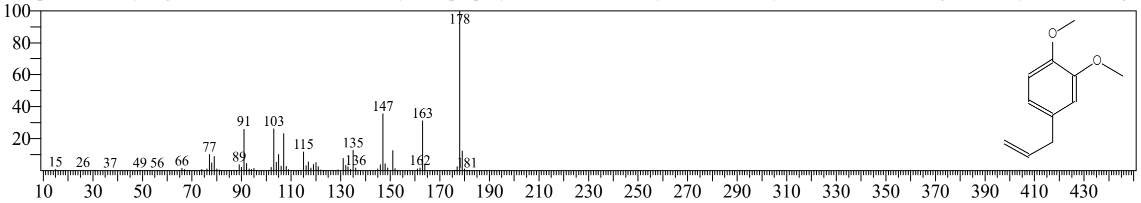

<< Target >>
Line#:18   R.Time:23.975(Scan#:4196) MassPeaks:17
RawMode:Averaged 23.970-23.980(4195-4197) BasePeak:192.10(6260)
BG Mode:Calc. from Peak Group 1 - Event 1 Scan

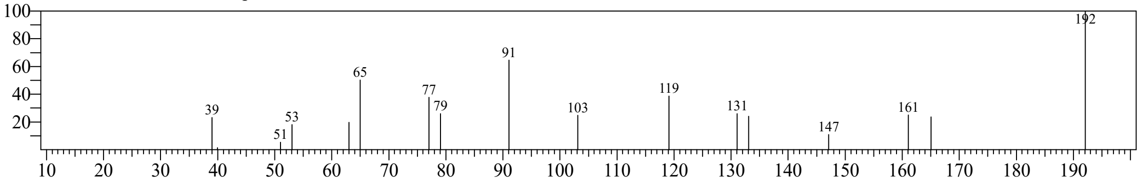

Hit#:1 Entry:21882 Library:NIST23s.lib
SI:78 Formula:C11H12O3 CAS:18312-21-5 MolWeight:192 RetIndex:1629
CompName:trans-Isomyristicin $$ 1,3-Benzodioxole, 4-methoxy-6-(1E)-1-propen-1-yl- $$ 1,3-Benzodioxole, 4-methoxy-6-(1E)-1-propenyl- $$ 1,3-Benzod

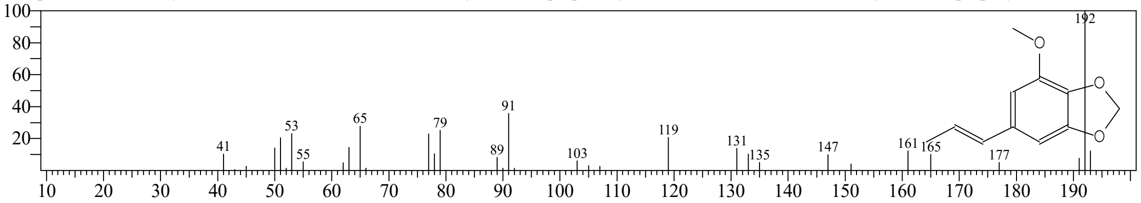

Hit#:2 Entry:50402 Library:NIST23-1.lib
SI:77 Formula:C11H12O3 CAS:607-91-0 MolWeight:192 RetIndex:1537
CompName:1,3-Benzodioxole, 4-methoxy-6-(2-propenyl)- $$ Benzene, 5-allyl-1-methoxy-2,3-(methylenedioxy)- $$ Myristicin $$ 5-Allyl-1-methoxy-2,3-(n

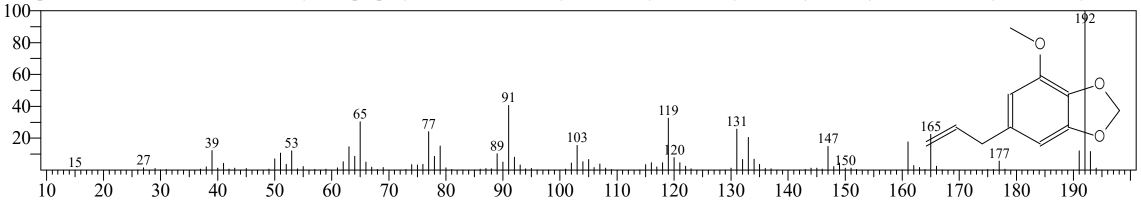

Hit#:3 Entry:50406 Library:NIST23-1.lib
SI:71 Formula:C11H12O3 CAS:194609-21-7 MolWeight:192 RetIndex:1575
CompName:Isocroweacin,E-

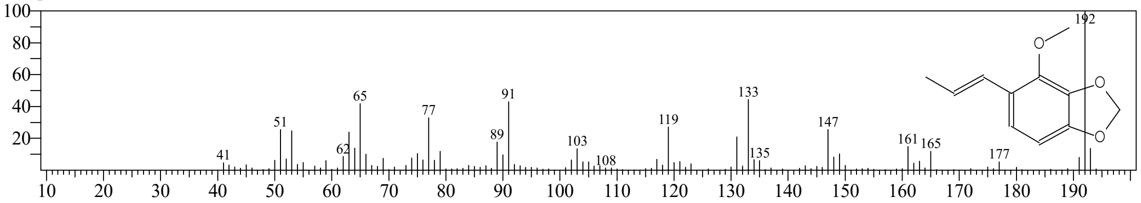

Hit#:4 Entry:50399 Library:NIST23-1.lib
SI:71 Formula:C11H12O3 CAS:484-34-4 MolWeight:192 RetIndex:1496
CompName:Croweacin

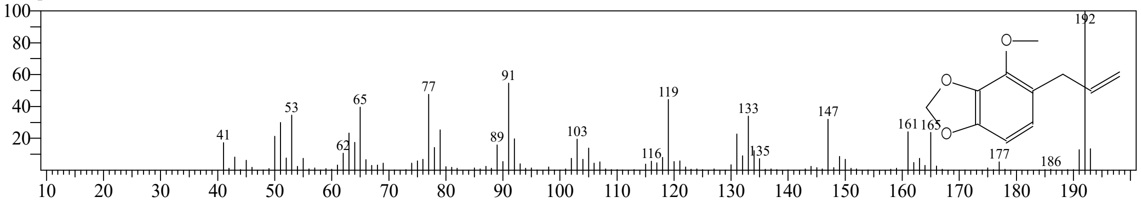

Hit#:5 Entry:50408 Library:NIST23-1.lib
SI:69 Formula:C11H12O3 CAS:18607-93-7 MolWeight:192 RetIndex:1529
CompName:Asaricin

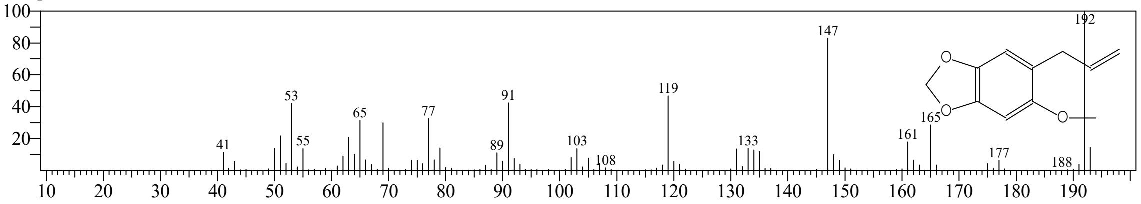

<< Target >>
Line#:19   R.Time:24.580(Scan#:4317) MassPeaks:11
RawMode:Averaged 24.575-24.585(4316-4318) BasePeak:208.10(5397)
BG Mode:Calc. from Peak Group 1 - Event 1 Scan

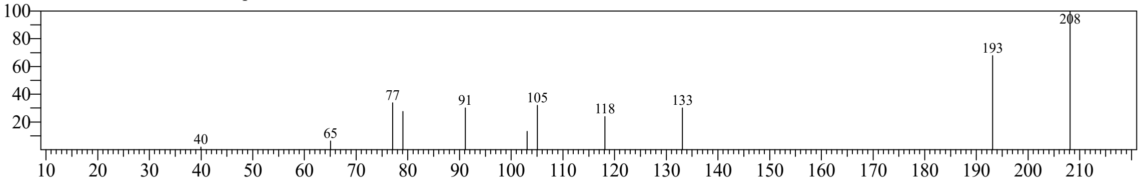

Hit#:1 Entry:67193 Library:NIST23-1.lib
SI:72 Formula:C12H16O3 CAS:487-12-7 MolWeight:208 RetIndex:1647
CompName:Isoelemicin $$ 1,2,3-Trimethoxy-5-(1-propenyl)benzene # $$ Benzene, 5(1-propenyl)-1,2,3-trimethoxy $$

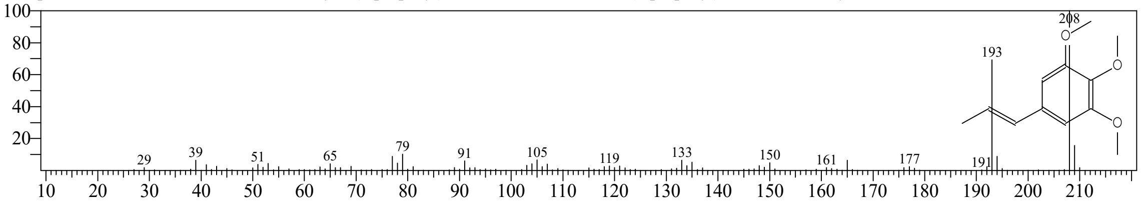

Hit#:2 Entry:67185 Library:NIST23-1.lib
SI:71 Formula:C12H16O3 CAS:5273-85-8 MolWeight:208 RetIndex:1647
CompName:Benzene, 1,2,3-trimethoxy-5-(1-propenyl)-, (E)- $$ Benzene, 1,2,3-trimethoxy-5-propenyl-, (E)- $$ Isoelemicin $$

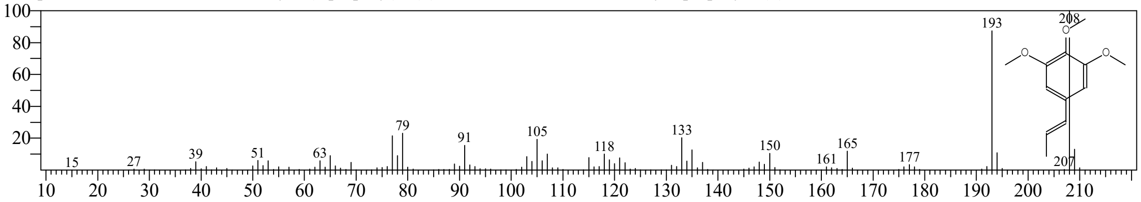

Hit#:3 Entry:25892 Library:NIST23s.lib
SI:70 Formula:C12H16O3 CAS:487-11-6 MolWeight:208 RetIndex:1550
CompName:Benzene, 1,2,3-trimethoxy-5-(2-propenyl)- $$ Benzene, 5-allyl-1,2,3-trimethoxy- $$ Elemicin $$ 3,4,5-Trimethoxyallylbenzene $$ 5-Allyl-1,2,3

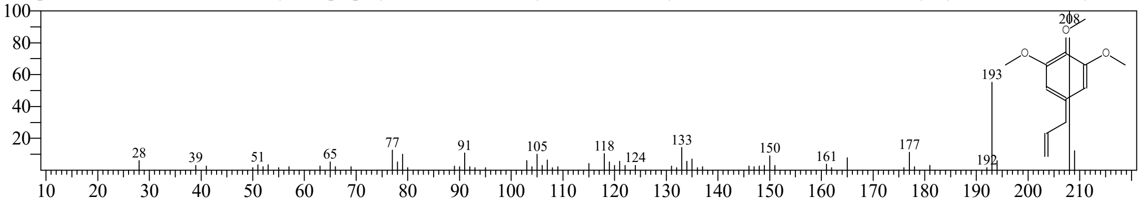

Hit#:4 Entry:25817 Library:NIST23s.lib
SI:67 Formula:C11H12O4 CAS:2316-26-9 MolWeight:208 RetIndex:1940
CompName:3,4-Dimethoxycinnamic acid $$ Dimethyl caffeic acid $$ 2-Propenoic acid, 3-(3,4-dimethoxyphenyl)- $$ Caffeic acid dimethyl ether $$ Cinnam

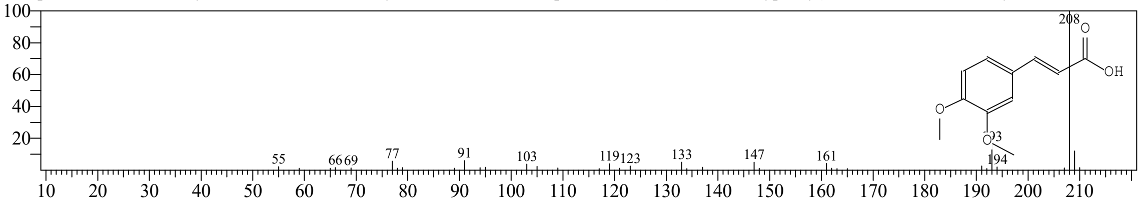

Hit#:5 Entry:66867 Library:NIST23-1.lib
SI:67 Formula:C11H12O4 CAS:14737-89-4 MolWeight:208 RetIndex:1940
CompName:2-Propenoic acid, 3-(3,4-dimethoxyphenyl)-, (E)- $$ (2E)-3-(3,4-Dimethoxyphenyl)-2-propenoic acid $$ (E)-3,4-Dimethoxycinnamic acid $$

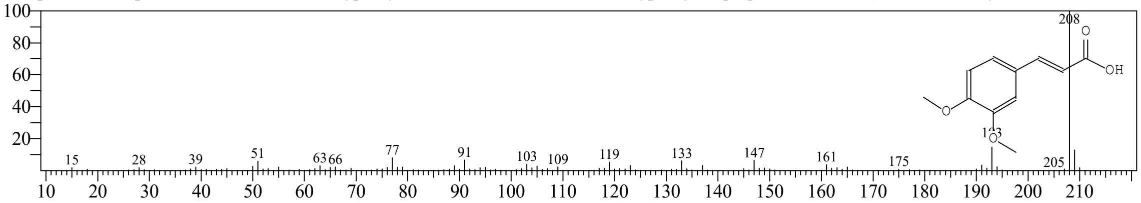

<< Target >>
Line#:20 R.Time:42.145(Scan#:7830) MassPeaks:5
RawMode:Averaged 42.140-42.150(7829-7831) BasePeak:91.10(10768)
BG Mode:Calc. from Peak Group 1 - Event 1 Scan

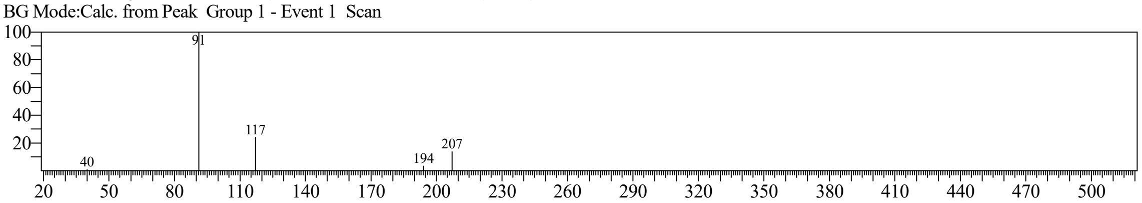

Hit#:1 Entry:65437 Library:NIST23-1.lib
SI:79 Formula:C9H9N3OS CAS:0-00-0 MolWeight:207 RetIndex:2213
CompName:4H-1,2,4-triazol-3-ol, 5-[(phenylmethyl)thio]- $$ 5-(benzylsulfanyl)-4H-1,2,4-triazol-3-ol $$

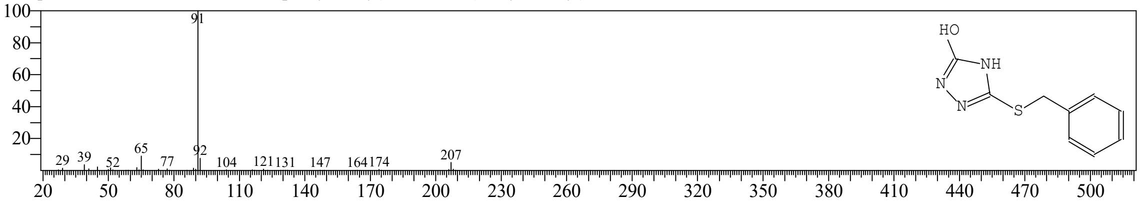

Hit#:2 Entry:163412 Library:NIST23-1.lib
SI:78 Formula:C16H15NO4 CAS:63909-29-5 MolWeight:285 RetIndex:2549
CompName:Benzene, 2-benzyloxy-1-methoxy-4-(2-nitroethenyl)- $$ 2-(Benzyloxy)-1-methoxy-4-[(E)-2-nitroethenyl]benzene # $$

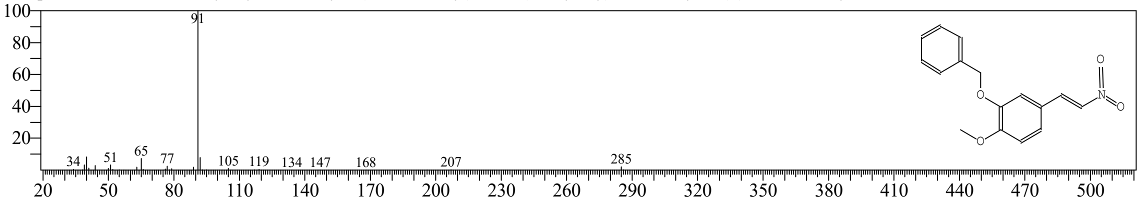

Hit#:3 Entry:163413 Library:NIST23-1.lib
SI:76 Formula:C16H15NO4 CAS:74810-83-6 MolWeight:285 RetIndex:2434
CompName:Benzene, 2-methoxy-1-(2-nitroethenyl)-3-(phenylmethoxy)- $$ 1-(Benzyloxy)-2-methoxy-3-[(E)-2-nitroethenyl]benzene # $$

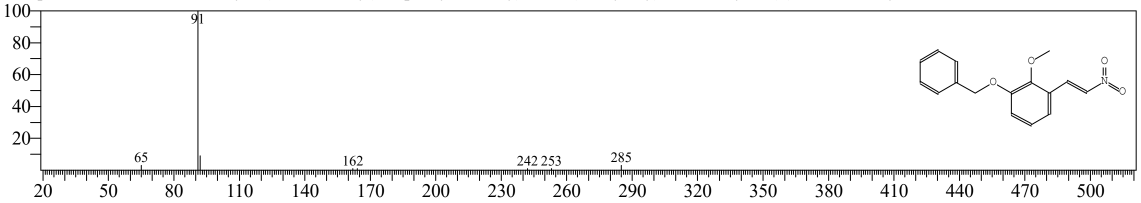

Hit#:4 Entry:87058 Library:NIST23-2.lib
SI:76 Formula:C31H32O5Si CAS:0-00-0 MolWeight:512 RetIndex:3610
CompName:3,4,5-Tris(phenylmethoxy)benzoic acid, TMS $$ Benzoic acid, 3,4,5-tris(phenylmethoxy)-, trimethylsilyl ester $$

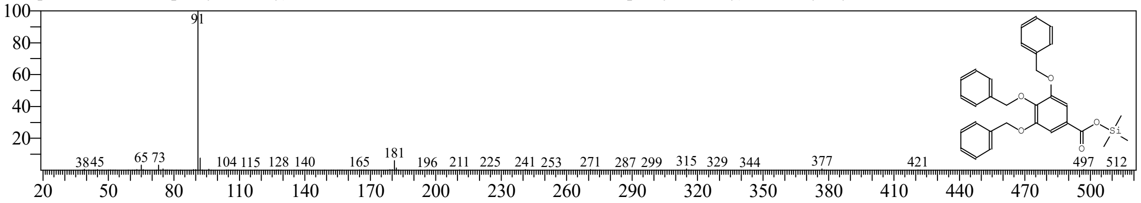

Hit#:5 Entry:170232 Library:NIST23-1.lib
SI:76 Formula:C16H15ClO3 CAS:63007-66-9 MolWeight:290 RetIndex:2264
CompName:Benzofenac methyl ester $$ Methyl [4-(benzyloxy)-3-chlorophenyl]acetate # $$

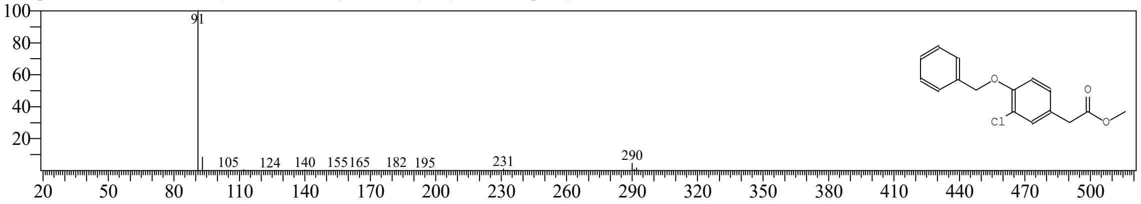

<< Target >>
Line#:21 R.Time:43.495(Scan#:8100) MassPeaks:9
RawMode:Averaged 43.490-43.500(8099-8101) BasePeak:277.10(18972)
BG Mode:Calc. from Peak Group 1 - Event 1 Scan

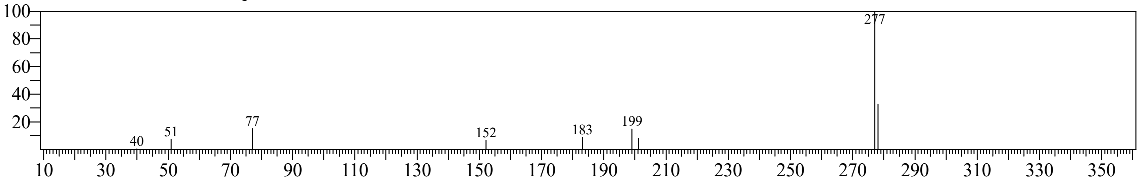

Hit#:1 Entry:36113 Library:NIST23s.lib
SI:88 Formula:C18H15OP CAS:791-28-6 MolWeight:278 RetIndex:2583
CompName:Triphenylphosphine oxide $$ Phosphine oxide, triphenyl- $$ Triphenyl phosphorus oxide $$ (C6H5)3P=O $$ Triphenylphosphanoxid $$ Triphe

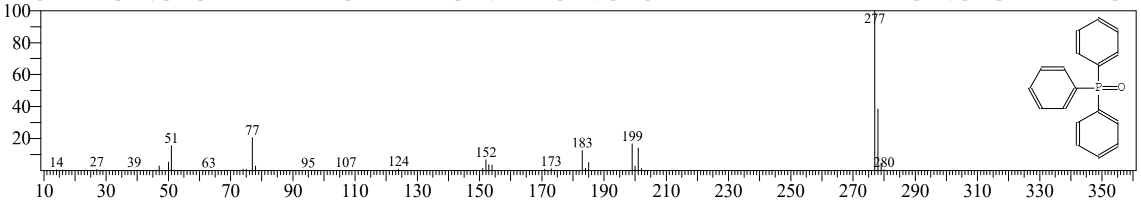

Hit#:2 Entry:4053 Library:NIST23-2.lib
SI:80 Formula:C21H23O3P CAS:0-00-0 MolWeight:354 RetIndex:2542
CompName:Dimethoxymethyl-hydroxy-triphenyl phosphide $$ (Dimethoxymethyl)(hydroxy)triphenylphosphorane # $$

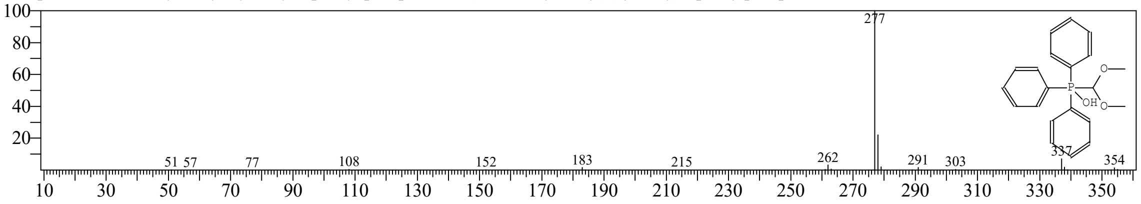

Hit#:3 Entry:61806 Library:NIST23-2.lib
SI:75 Formula:C22H22BrO2P CAS:1530-45-6 MolWeight:428 RetIndex:2739
CompName:(Carbethoxymethyl)-triphenylphosphonium bromide $$ Carboethoxymethyltriphenylphosphonium bromide $$ Ethoxycarbonylmethyltriphenylp

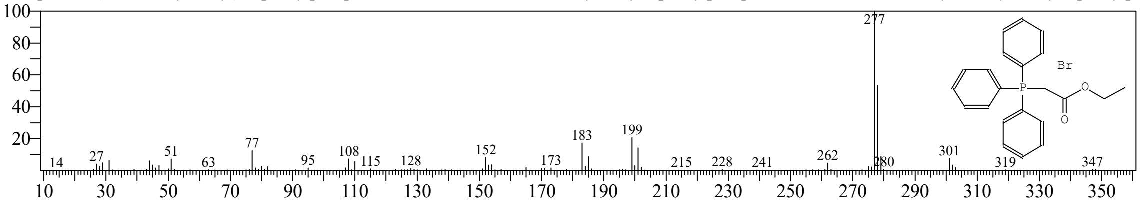

Hit#:4 Entry:189577 Library:NIST23-1.lib
SI:75 Formula:C19H17N2P CAS:15990-54-2 MolWeight:304 RetIndex:2629
CompName:Formaldehyde, (triphenylphosphoranylidene)hydrazone $$ Phosphorane, (methylenehydrazono)triphenyl- $$

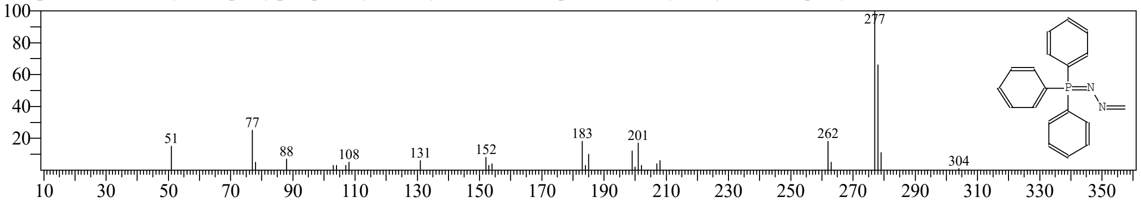

Hit#:5 Entry:174065 Library:NIST23-1.lib
SI:73 Formula:C15H19NO3S CAS:0-00-0 MolWeight:293 RetIndex:2445
CompName:(E)-7-Benzylidene-1-azabicyclo[3.2.1]octan-5-yl methanesulfonate

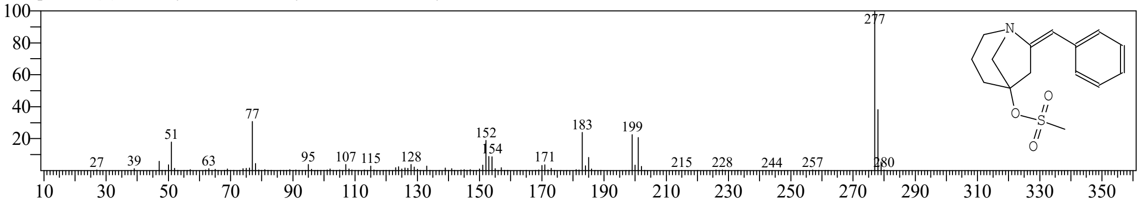

<< Target >>
Line#:22   R.Time:46.520(Scan#:8705) MassPeaks:3
RawMode:Averaged 46.515-46.525(8704-8706) BasePeak:57.10(1162)
BG Mode:Calc. from Peak Group 1 - Event 1 Scan

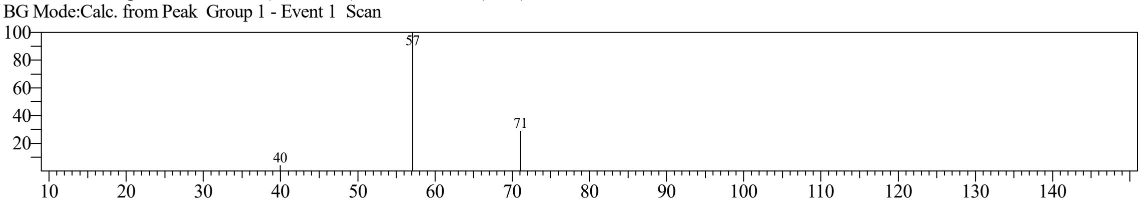

Hit#:1 Entry:9021 Library:NIST23-1.lib
SI:87 Formula:C6H10O3 CAS:123-62-6 MolWeight:130 RetIndex:921
CompName:Propanoic acid, anhydride $$ Methylacetic anhydride $$ Propanoic anhydride $$ Propionic acid anhydride $$ Propionic anhydride $$ Propiony

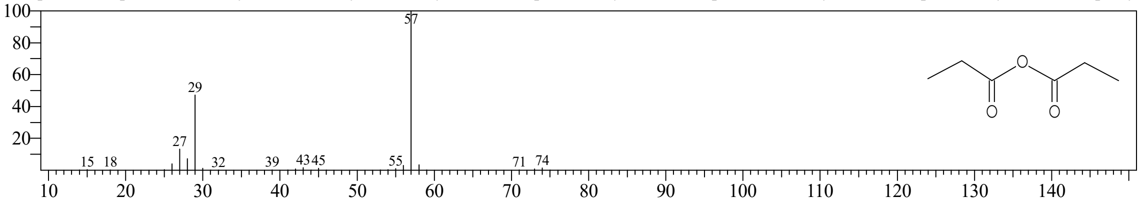

Hit#:2 Entry:4051 Library:NIST23s.lib
SI:85 Formula:C6H10O2 CAS:4437-51-8 MolWeight:114 RetIndex:805
CompName:3,4-Hexanedione $$ Bipropionyl $$ 3,4-Hexandione $$

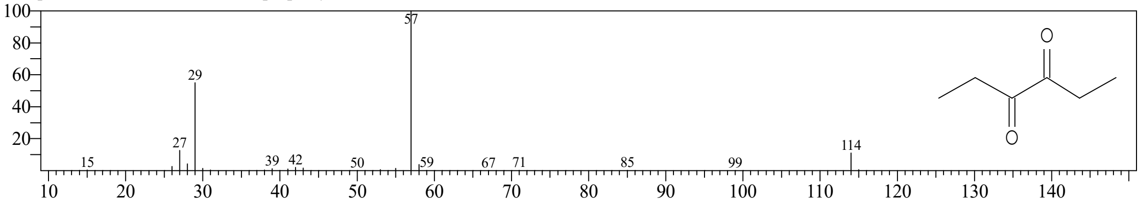

Hit#:3 Entry:122851 Library:NIST23-1.lib
SI:84 Formula:C18H38 CAS:62850-21-9 MolWeight:254 RetIndex:1465
CompName:Hexane, 3,4-bis(1,1-dimethylethyl)-2,2,5,5-tetramethyl- $$ 3,4-Ditert-butyl-2,2,5,5-tetramethylhexane # $$ 1,1,2,2-Tetra-t-butylethane $$

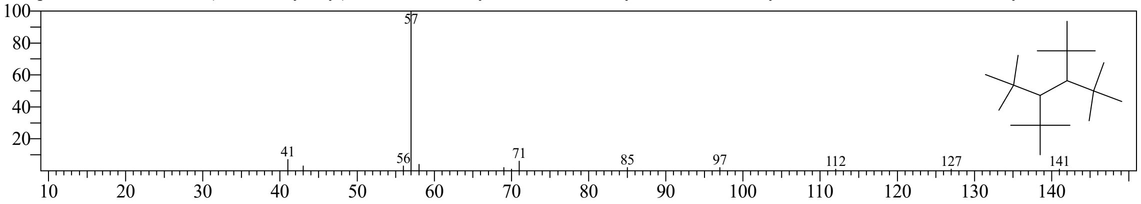

Hit#:4 Entry:320 Library:NIST23-1.lib
SI:83 Formula:C3H9Al CAS:75-24-1 MolWeight:72 RetIndex:443
CompName:Trimethylaluminum $$ (CH3)3Al $$ Aluminum, trimethyl- $$ Trimethylalane $$ Trimethylaluminium $$ UN 1103 $$

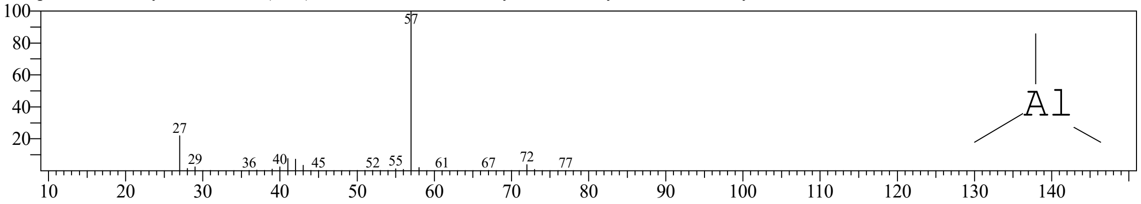

Hit#:5 Entry:1228 Library:NIST23s.lib
SI:82 Formula:C4H8O2 CAS:5077-67-8 MolWeight:88 RetIndex:769
CompName:1-Hydroxy-2-butanone $$ 2-Butanone, 1-hydroxy- $$

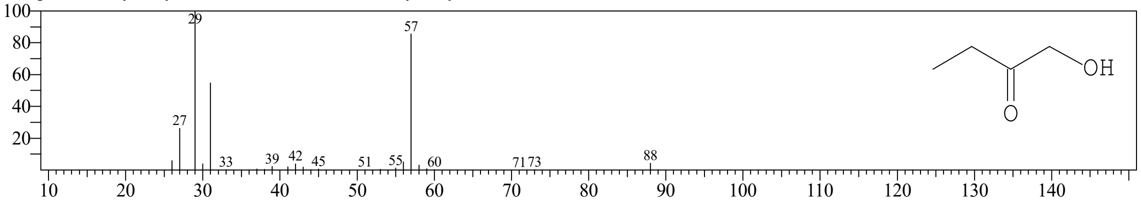

Supplement: Supplementary file 1 [file pharmaceuticals-19-00233-s001.zip › pharmaceuticals-4066640-Supplementary File S1.pdf]
